# The Crucial Role of LTTR_0390 in *Burkholderia gladioli* BBB-01 in Orchestrating Antibiotic Production, Quorum-Sensing Responses, and Pathogenicity on Mushrooms

**DOI:** 10.1021/acs.jafc.5c12363

**Published:** 2026-03-30

**Authors:** Ali Diyapoglu, Yu-You Liu, Alican Abay, Kuan-Hung Lin, I-Wen Lo, Chi-Fon Chang, Yi-Ping Huang, Chi-Ting Chung, Tsung-Lin Li, Menghsiao Meng

**Affiliations:** † Graduate Institute of Biotechnology, 34916 National Chung Hsing University, Taichung 40227, Taiwan; ‡ Molecular and Biological Agricultural Sciences, Taiwan International Graduate Program, Academia Sinica and National Chung Hsing University, Taipei 11529, Taiwan; § 684260 Agricultural Biotechnology Research Center, Academia Sinica, Taipei 11529, Taiwan; ∥ Genomics Research Center, Academia Sinica, Taipei 11529, Taiwan; ⊥ National Research Institute of Chinese Medicine, Ministry of Health and Welfare, Taipei 112026, Taiwan; # Bachelor Program in Biotechnology, 34916 National Chung Hsing University, Taichung 40227, Taiwan; ∇ Biotechnology Center, National Chung Hsing University, Taichung 40227, Taiwan

**Keywords:** *Burkholderia gladioli*, antibiotic production, secondary metabolites, quorum sensing, LysR-type transcription regulator, mushrooms soft rot

## Abstract

*Burkholderia gladioli* strain BBB-01, classified as *B. gladioli* pv *agaricicola*, displays broad antagonism against diverse pathogens. We investigated the regulatory role of the LysR-type transcriptional regulator LTTR_0390 and its potential involvement in pathogenicity toward the cultivated button mushroom (*Agaricus bisporus*). Using insertional mutagenesis, metabolite profiling, transcriptomic analysis, and phenotypic assays, we found that disruption of the *lttr_0390* gene extensively reprograms secondary metabolism, for example, upregulating the gladiostatin biosynthetic gene cluster while downregulating the gladiolin cluster. Although this gene disruption did not affect production of quorum-sensing (QS) autoinducers, the mutant exhibited reduced QS-linked behaviors, including motility and biofilm formation. Additionally, disruption of *lttr_0390* markedly diminished soft rot symptoms on *A. bisporus* fruiting bodies. Together, these findings identify LTTR_0390 as a global regulator that coordinates secondary metabolism and QS-linked behaviors while decoupling antifungal activity from mushroom damage.

## Graphical abstract

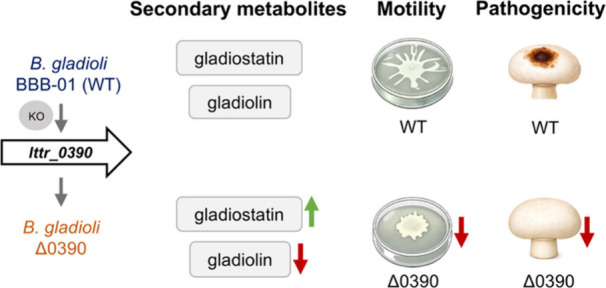

## Introduction

Given the global rise of antibiotic-resistant pathogens and their serious threat to human health, the discovery of new, effective antibiotics remains a top priority in research. For most of the past century, antibiotics have primarily been derived from the genus *Streptomyces*. However, in recent decades, focus has increasingly shifted to *Burkholderia*, because their genomes harbor numerous cryptic biosynthetic gene clusters (BGCs) with the potential to yield novel antibiotics.^1,2^

Members of the genus *Burkholderia* are Gram-negative, rod-shaped, motile, and nonspore-forming bacteria widely distributed in various ecological niches.^3,4^ Some strains form mutualistic or pathogenic relationships with specific plant and animal hosts. Compared to typical bacteria, *Burkholderia* have large genomes, consisting of two circular chromosomes and 1–3 large plasmids, with a combined base pair count of approximately 8 million. The first chromosome mainly carries housekeeping genes responsible for metabolism and growth, while the second chromosome is rich in BGCs, and the plasmids often harbor pathogenicity genes.

Within the genus *Burkholderia*, *B. gladioli* stands out for its remarkable ecological versatility, inhabiting diverse environments and engaging in a wide range of interactions, from causing diseases in plants, fungi, and humans to forming symbiotic relationships with insects. This species is subdivided into four pathovars (pv): *B. gladioli* pv *alliicola*, *B. gladioli* pv *gladioli*, *B. gladioli* pv *agaricicola*, and *B. gladioli* pv *cocovenenans*, all of which are linked with economic losses in crop production or potentially threatening human health.^5–7^ For example, *B. gladioli* pv *agaricicola* causes soft rot in edible mushrooms, such as *Agaricus bisporus, Agaricus bitorquis, Hypsizygus marmoreus, Pleurotus ostreatus*, and *Pleurotus eryngii*.^8,9^ Outbreaks lead to rapid tissue decay and cavity formation, threatening the global multibillion-dollar mushroom industry and accounting for ∼25% average annual production loss in Western countries.^9,10^

A handful of secondary metabolites with diverse bioactivities have been identified from the culture of *B. gladioli* strains. Icosalide, a lipopeptide with dual fatty acids, is synthesized by *B. gladioli* Lv-StA, a symbiont found in the reproductive tract of the beetle *Lagria villosa*, and it can protect the beetle’s eggs from fungal infections.^11^ This beetle symbiont also produces gladiofungin, a polyketide featuring a unique glutarimide structure, which inhibits *Penicillium notatum* and *Sporobolomyces salmonicolor*,^12^ and sinapigladioside, an antifungal isothiocyanate.^13^ Gladiolin, a polyketide produced by *B. gladioli* BCC0238, exhibits strong inhibitory activity against drug-resistant *Mycobacterium tuberculosis*.^14^ Gladiostatin (syn. Gladiofungin), produced also by the strain BCC0238, exhibits cytotoxic activity against various human cancer cell lines.^15^ Bongkrekic acid, a polyketide produced by *B. gladioli* pv *cocovenenans*, is a highly toxic tricarboxylic acid that blocks the ADP/ATP translocase, disrupting cellular energy metabolism.^16^ Toxoflavin is a potent phytotoxin with broad-spectrum antifungal activity. Its production by *B. gladioli* HDXY-02, an endophytic bacterium of the herb *Lycoris aurea*, protects the herb from the infection of *Aspergillus fumigatus*.^17^

The number of secondary metabolites found in *Burkholderia* so far is lower than the number of BGCs predicted by bioinformatics analysis of sequenced genomes from this genus. This gap indicates that many predicted BGCs are probably silent or hidden under standard laboratory culture conditions.^18,19^ Consequently, these functionally uncharacterized BGCs in *Burkholderia* genomes hold a large potential for natural products with therapeutic uses. Changing culture conditions or making specific gene modifications, like deleting or overexpressing regulatory genes, might activate these silent BGCs, showing the importance of continued research.^20^

LysR-type transcriptional regulators (LTTRs) are a large family of bacterial regulatory proteins that control various biological functions, particularly by regulating gene expression in response to environmental and cellular signals. The regulated functions include secondary metabolite production, stress responses, motility, virulence, biofilm formation, and metabolism.^21,22^ In terms of antibiotic production, disrupting specific LTTR genes may increase the production of certain antibiotics while reducing that of others; for example, the deletion of *scmR*, a QS-controlled LTTR, in *Burkholderia thailandensis* E264 greatly enhances the production of malleilactone, bactobolin, thailandamide, capistruin, and burkholdac, but reduces that of 4-hydroxy-3-methyl-2-alkylquinoline.^23^ Likewise, in *Burkholderia cenocepacia* H111, the LTTR Bcal3178 operates downstream of the QS signaling pathway, regulating QS-controlled phenotypes such as biofilm formation and protease production.^24^

We recently isolated a *B. gladioli* strain from the surface of rice shoots, named BBB-01. Pangenomic analysis suggested that the strain BBB-01 belongs to the pv *agaricicola*.^25^ While this strain was avirulent to rice, it could inhibit the mycelial growth of several phytopathogenic fungi of rice, including *Magnaporthe oryzae, Gibberella fujikuroi*, and *Sarocladium oryzae*, through the release of water-diffusible substances and volatile organic compounds (VOCs) such as dimethyl disulfide (DMDS) and 2,5-dimethylfuran.^25^ *B. gladioli* BBB-01 also emits *S*-methyl thioacetate (SMT), which exhibits stronger nematicidal activity via fumigation against root-knot nematode *Meloidogyne incognita* compared to the renowned pesticidal fumigant DMDS.^26^

Here, we purify and identify the antibiotics produced by *B. gladioli* BBB-01, link these compounds to their corresponding BGCs, and show that an ScmR-like LysR-type regulator, LTTR_0390 (encoded by *lttr_0390*), coordinates their production while also modulating QS-dependent behaviors and pathogenic traits. We also test whether BBB-01 harms the cultivated button mushroom, *A. bisporus*. The orchestrating role of LTTR_0390 is detailed herein.

## Materials and Methods

### Microorganisms

The bacterial strains used in this study are summarized in Table S1. Some of these strains, including *B. gladioli* BBB-01, *Burkholderia cepacia* Xitou, *Staphylococcus aureus, Pseudomonas aeruginosa*, and *Klebsiella pneumoniae*, were multidrug-resistant despite not being isolated from clinical patients. Notably, the *P. aeruginosa* and *S. aureus* strains exhibit resistance to a wide range of antibiotics, including ampicillin, apramycin, carbenicillin, cefotaxime, chloramphenicol, erythromycin, kanamycin, nourseothricin, oxacillin, streptomycin, and tetracycline.

### Genetic Manipulation

Plasmid pBBR1-Rha-ETh1h2e_yi23-kan, used to facilitate the homologous recombination in *Burkholderia*,^27^ was purchased from Addgene (Watertown, MA). The kanamycin-resistant gene (*kan* ^*R*^) in pBBR1-Rha-ETh1h2e_yi23-kan was replaced by the tetracycline-resistant gene (*tet* ^*R*^) cropped from plasmid pBR322 using the NEBuilder HiFi DNA assembly master mix (NEB, Ipswich, MA), resulting in plasmid pBBR1-Rha-ETh1h2e_yi23-tet. Besides, the recombinase operon RecETh1h2e_YI23_ in pBBR1-Rha-ETh1h2e_yi23-kan was substituted with the *lttr_0390* ORF, resulting in plasmid pBBR-0390. A DNA fragment containing *tet* ^*R*^ with the preceding Neo/Kan promoter was amplified from pBBR1-Rha-ETh1h2e_yi23-tet by PCR; furthermore, it was flanked with two ∼150 nt sequences, homologous to the 5′ and 3′ regions of *lttr_0390*, respectively, by overlap extension PCR to become the fragment *lttr_0390::tet* ^*R*^ that was used later for the disruption of the chromosomal *lttr_0390*.

To disrupt chromosomal *lttr_0390*, *B. gladioli* BBB-01 was first transformed with pBBR1-Rha-ETh1h2e_yi23-kan by the electroporation method using the MicroPulser Electroporator (Bio-Rad Laboratories, Hercules, CA), with the condition set to Ec2 (2.5 kV). The overnight culture of the resulting strain, cultivated in Lysogeny broth (LB) medium supplemented with kanamycin (final concentration: 100 μg/mL) at 28 °C, was diluted 30-fold and then continuously grown in the same medium until the optical density at 600 nm (OD_600_) reached 0.5. Rhamnose was added to a final concentration of 1 mg/mL to induce the recombinase expression for 1 h. The cells were then introduced with the overlap extension PCR product *lttr_0390::tet* ^*R*^ using the same electroporation method. The transformants that resisted 100 μg/mL tetracycline and kanamycin were selected. A plasmid curing process was conducted to remove pBBR1-Rha-ETh1h2e_yi23-kan in the transformants by growing the cells in an LB/tetracycline medium at 37 °C for several passages until the transformants were no longer resistant to kanamycin. The tetracycline-resistant and kanamycin-sensitive transformants were considered potentially *lttr_0390* knockout strains. The targeted disruption of chromosomal *lttr_0390* was confirmed by PCR using two primer pairs, Bga_0390F with Bga_0390R and pNeo/Kan with Bga_0390R, and the confirmed knockout strain is thus termed *B. gladioli* Δ0390 strain. All primers used in this study are listed in Table S2. For complementary assays, the plasmid pBBR-0390 was introduced into *B. gladioli* Δ0390 strain by the same electroporation method to obtain the complemented strain *B. gladioli* Δ0390/pBBR-0390.

### Antagonist Activity Assays

The double-layer assay was performed to determine the ability of the *B. gladioli* strains to inhibit the growth of challenged bacteria.^28^ A 3 μL aliquot of the overnight culture of *B. gladioli* was dropped onto a 9 cm Petri dish agar plate containing 0.4% potato infusion powder, 2% dextrose, and 1.5% agar (PDA) and incubated at 28 °C for 4 days. The bacterial cells grown on the PDA plate were then killed by chloroform vapor in a sealed chamber for 5 h. After fumigation, 10 mL of LB or nutrient broth (NB) medium containing 0.75% agar was poured onto a PDA plate. The challenged bacterium or yeast was then evenly spread across the plate using a cotton swab soaked in a culture with an OD_600_ of 0.05. The double-layer plate was then incubated at 28 or 37 °C for 1–2 days.

The confrontation assay was performed to determine the ability of the *B. gladioli* BBB-01 strain to suppress mycelium growth of pathogenic molds. A 1 cm diameter agar plug full of the challenged mycelium was placed at the center of a 9 cm Petri dish of PDA and incubated at 28 °C for 5 days. Then, a 3 μL culture of the *B. gladioli* strain was dropped onto a spot 2.5 cm away from the central fungal plug. The plate was further incubated at 28 °C for 10 days.

### Pathogenicity Assay on *A. bisporus*

Fresh mushrooms purchased from local markets were surface-cleaned by gently wiping with a cotton swab moistened with sterile water. Overnight LB cultures of the test strains were adjusted to an OD_600_ of 0.25 (≈10^8^ CFU/mL) and subsequently serially diluted to 10^6^ and 10^4^ CFU/mL. A 20 μL aliquot of each dilution was spotted onto the surface of each mushroom cap, while LB medium alone served as the negative control. The inoculated mushrooms were placed in sealed plastic boxes and incubated at 25 °C for 2–3 days.

### Secondary Metabolite Extraction and Purification

The wild-type (WT), Δ0390, and Δ0390/pBBR-0390 strains of *B. gladioli* BBB-01 were respectively streaked on the entire surface of PDA plates and incubated at 28 °C for 4 days. After removing the cell mass using a cell scraper, the agar was diced into small cubes and mixed with an equal volume of ethyl acetate (EA). The mixture was agitated in an orbital shaker at 200 rpm, room temperature, for 2 days. The extraction solution was concentrated under reduced pressure using a rotary evaporator. The dry residue was dissolved in ethanol at a volume ratio of 1:50 relative to the original EA extract.

The secondary metabolites in ethanol were fractionated using Agilent 1200 reverse-phase high-pressure liquid chromatography (RP-HPLC) (Santa Clara, CA). A 100 μL ethanol solution was injected into an Ascentis C18 column (25 cm × 10 mm × 5 μm, Burlington, MA). Elution was performed using mobile phases consisting of 0.1% (v/v) formic acid aqueous solution (A) and 0.1% (v/v) formic acid-containing acetonitrile (B) at a flow rate of 1.5 mL/min, with the detection of absorbance at 220 nm. The elution steps were set as follows: 0–5 min: 2–50% B; 5–60 min, 50–100% B; 60–61 min, 100–2% B; 61–65 min, 2% B. The collected fractions were dried in liquid nitrogen and then lyophilized using a vacuum freeze-dryer.

### Metabolite Identification and Structural Characterization

The molecular weights of purified metabolites were confirmed using a linear ion trap mass spectrometer (Thermo Scientific LTQ XL, Waltham, MA) coupled to an Agilent 1200 HPLC equipped with an Ascentis C18 column. Mobile phases and elution conditions were as described above. Data were acquired in full-scan mode (*m*/*z* 250–2000) in both ESI+ and ESI–.

The structures of the purified secondary metabolites were elucidated using 1D and 2D nuclear magnetic resonance (NMR) analysis. Purified fractions were dissolved in methanol-*d*_*4*_ (CD_3_OD) (F3, F4/F5, and F6), DMSO-*d*_*6*_ (F7), and pyridine-*d*_*5*_ (F3 degradation product) and subjected to analysis with ^1^H, ^13^C, correlation spectroscopy (COSY), total correlation spectroscopy (TOCSY), heteronuclear single-quantum correlation spectroscopy (HSQC), heteronuclear multiple-bond correlation spectroscopy (HMBC), and nuclear Overhauser effect spectroscopy (NOESY). All spectra were acquired on a Bruker AVANCE III 600 spectrometer in the Genomics Research Center, Academia Sinica, Taiwan.

### Minimum Inhibitory Concentration (MIC) Assay

The antagonistic bacteria and yeasts were cultured in LB or NB medium, respectively, and incubated at 37 °C for 3 h. The culture was adjusted to an optical density at 600 nm of approximately 0.1, then diluted 100-fold in fresh LB medium for bacteria or 50-fold in fresh NB medium for yeasts. In a 96-well plate, 100 μL of the diluted culture was added to each well, followed by an equal volume of the corresponding medium containing serially diluted gladiostatin. The plate was sealed with a breathable film and incubated at 37 °C for 24 h. The MICs of gladiostatin for each antagonist were determined based on OD_600_ readings, measured using a Multiskan GO UV/vis microplate spectrophotometer (Thermo Fisher Scientific, Waltham, MA).

### Volatilome Analysis

As described previously, VOCs emitted by WT and Δ0390 strains were analyzed using solid-phase microextraction coupled with gas chromatography–mass spectrometry (GC-MS).^26^ Briefly, a 125 mL Erlenmeyer flask with 5 mL of LB agar was inoculated with 50 μL of bacterial culture and incubated at 28 °C for 3 days. A preconditioned 75 μm CAR/PDMS SPME fiber (Supelco, Bellefonte, PA) was inserted through parafilm and exposed to the headspace for 30 min. VOCs from blank medium served as controls. After extraction, the fiber was introduced into a Shimadzu QP2010 SE GC-MS system fitted with an RTx-5MS column. Helium was used as the carrier gas (1 mL/min). The GC oven was programmed as follows: 40 °C (5 min), ramped to 120 °C at 3 °C/min, to 180 °C at 4 °C/min, then to 280 °C at 20 °C/min, held for 5 min. VOCs were identified via mass spectral comparisons with the NIST20 GC-MS database.

### Bacterial Motility Assays

The overnight culture of the *B. gladioli* strains in LB was washed with fresh medium and adjusted to an OD_600_ of 0.25. A 3 μL aliquot of the cell suspension was positioned at the center of a 9 cm diameter LB agar plate and incubated at 28 or 37 °C for 5 days. The agar concentration was 0.25% (w/v) for the swimming motility assay and 0.5% (w/v) for the swarming motility assay.^29^

### Biofilm Formation Assay

The biofilm formation assay was adapted from a previous study.^30^ In brief, a 100 μL aliquot of the overnight bacterial culture in LB, with OD_600_ adjusted to 0.25, was added into a well of a 96-well microtiter plate and incubated at 28 °C for 1 day. After removal of the supernatant and washing with sterile water, 125 μL crystal violet solution [0.1% (w/v)] was added to the well and incubated at room temperature for 15 min. After removing the excess dye, 125 μL of 30% (v/v) acetic acid was added to the well, and the plate was incubated at room temperature for 15 min. The absorbance at 550 nm of the solution in the well was measured using a plate reader.

### Transcriptomic Analysis

A 100 μL overnight culture of the WT, Δ0390, or Δ0390/pBBR-0390 strain was transferred onto 15 cm diameter PDA plates and incubated at 28 °C for 2 days. Then, 2 mL of sterile water was added to each plate, and the cells were scraped off using a plastic scraper. The collected cells were washed twice with sterile water, followed by RNA extraction using 1.2 mL Trizol solution according to the manufacturer’s protocol (Thermo Fisher Scientific, Waltham, MA). Ribosomal RNA was depleted using the RiboMinus Bacteria 2.0 Transcriptome Isolation Kit (Thermo Fisher Scientific, Waltham, MA), leaving mRNA (mRNA) for subsequent analysis.

The mRNA was quantified via OD_260_ measurements using the ND-1000 spectrophotometer (Thermo Fisher Scientific, Waltham, MA) and assessed for quality with the Agilent 2100 Bioanalyzer and RNA 6000 Nano kit (Agilent Technologies, Santa Clara, CA). Complementary DNA libraries were prepared from the RiboMinus-treated RNA samples using the SureSelect XT HS2 mRNA Library Preparation Kit (Agilent Technologies) and purified with the HighPrep PCR Clean-up System (MagBio Genomics, Gaithersburg, MD). Library quality and concentration were determined using Qubit dsDNA Quantification Assay Kits and the Agilent 4200 TapeStation System (Agilent Technologies).

Sequencing was performed on the Illumina NovaSeq X Plus platform (Illumina, San Diego, CA). FASTQ files were generated using BCL Convert v4.2.4. Reads were mapped to the *B. gladioli* BBB-01 genome (NCBI RefSeq assembly: GCF_016698705.1) using Ensembl gene annotation database.^31^ Transcript assembly and quantification were conducted with StringTie software,^32^ and differential expression analysis was performed using the DESeq package software.^33^ The BGCs within the genomes of *B. gladioli* BBB-01 were predicted by the antiSMASH web server.^34^

### Statistical Analysis

Data were analyzed in GraphPad Prism 8.0.1. Differences among multiple groups were evaluated using one-way ANOVA followed by Tukey’s multiple-comparisons test. For pairwise comparisons (WT vs Δ0390) of LC–MS extracted-ion chromatogram (EIC) peak areas, a two-tailed Welch’s *t* test (unequal variances) was used. Data are presented as mean ± SD, and differences were considered statistically significant at *p* < 0.05.

## Results

### Antagonistic Activity of *B. gladioli* BBB-01

The double-layer assay was performed to determine the ability of the BBB-01 strain to inhibit the growth of challenged bacteria and yeasts (Figure 1). The BBB-01 strain exhibited strong inhibitory effects against *Bacillus cereus*, *S. aureus*, and *Ralstonia solanacearum* as demonstrated by the double-layer assay. While less effective, it also inhibited the growth of *B. cepacia* Xitou, *K. pneumoniae*, and *P. aeruginosa*. Among pathogenic yeasts, BBB-01 effectively suppressed *Candida glabrata* and *Candida parapsilosis* but not *Candida albicans*. In contrast, inhibition of *Escherichia coli* BL21 and *Saccharomyces cerevisiae* INVSc1 was weak and poorly defined, showing diffuse halos rather than clear inhibition zones (Figure 1A). Additionally, the confrontation assay confirmed that *B. gladioli* BBB-01 significantly suppressed the mycelium growth of rice blast pathogens *M. oryzae* and *Magnaporthe grisea* (Figure 1B).

**Figure 1:**
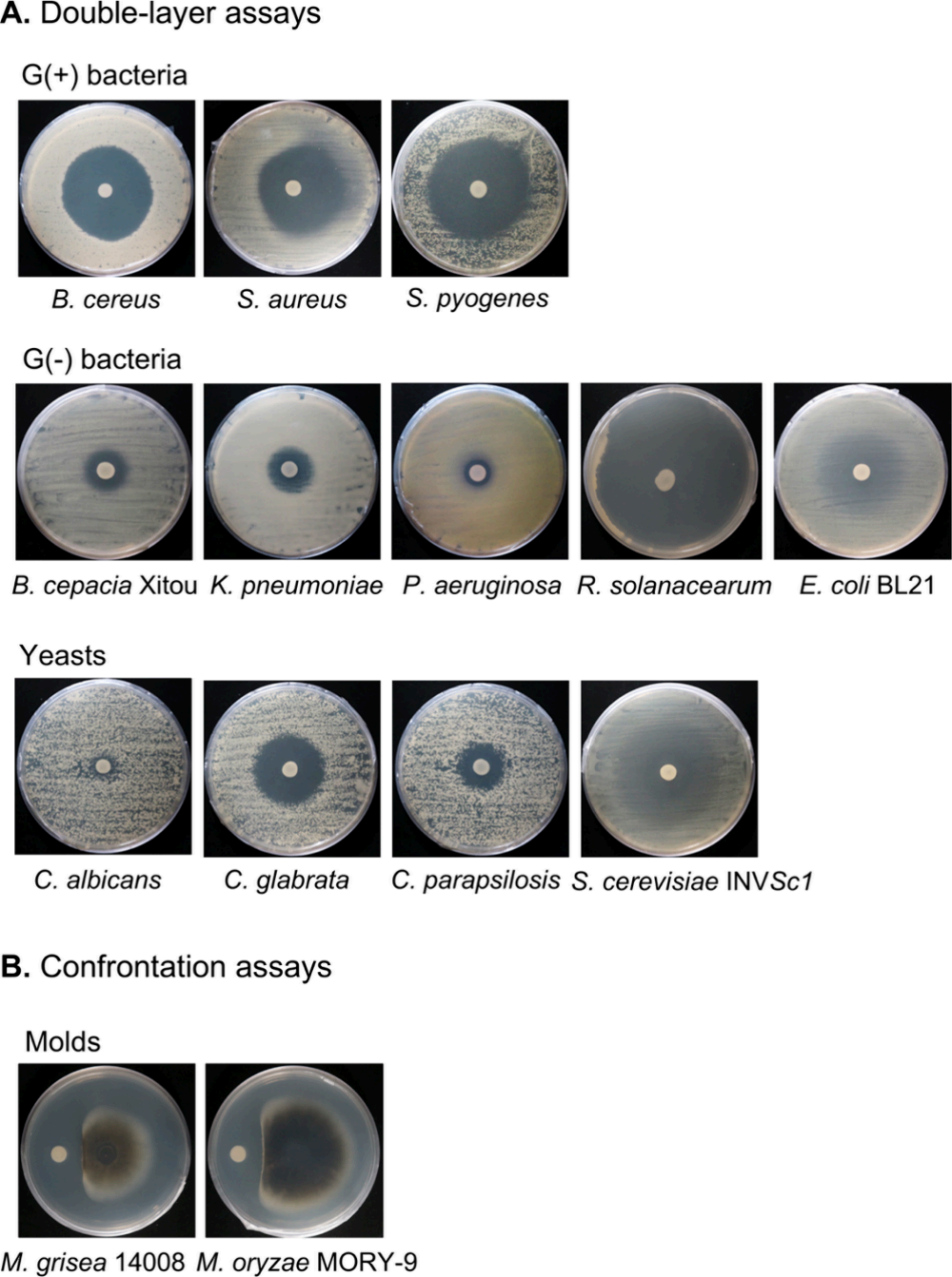
Antimicrobial activity of *B. gladioli* BBB-01. The antagonistic activity against the indicated bacteria and yeasts was evaluated by the double-layer assay (A), and activity against molds was assessed by the confrontation assay (B).

### Secondary Metabolites Produced by *B. gladioli* BBB-01

The genome of *B. gladioli* BBB-01 was analyzed by antiSMASH, which predicted 6 BGCs on chromosome 1 and 17 BGCs on chromosome 2 (Table S3). These BGCs include those responsible for producing previously characterized secondary metabolites, such as sinapigladioside, gladiolin (structurally similar to lagriene), icosalide, and gladiostatin. Secondary metabolites secreted by *B. gladioli* BBB-01 were collected from the cultivated agar, extracted with EA, and fractionated by RP-HPLC. Seven peaks were well resolved and designated F1–F7 (Figure 2A). LC–MS analysis revealed six distinct *m*/*z* features because F4 and F5 showed nearly identical MS behavior and were therefore treated as a combined F4/F5 feature; the observed *m*/*z* values were 836.73 (F1), 557.25 (F2), 506.24 (F3), 761.70 (F4/F5), 779.17 (F6), and 779.71 (F7) (Figure S1). For confirmation, we also performed high-resolution mass spectrometry (HRMS) analysis for F3 and F6 (Figure S2A,B).

**Figure 2:**
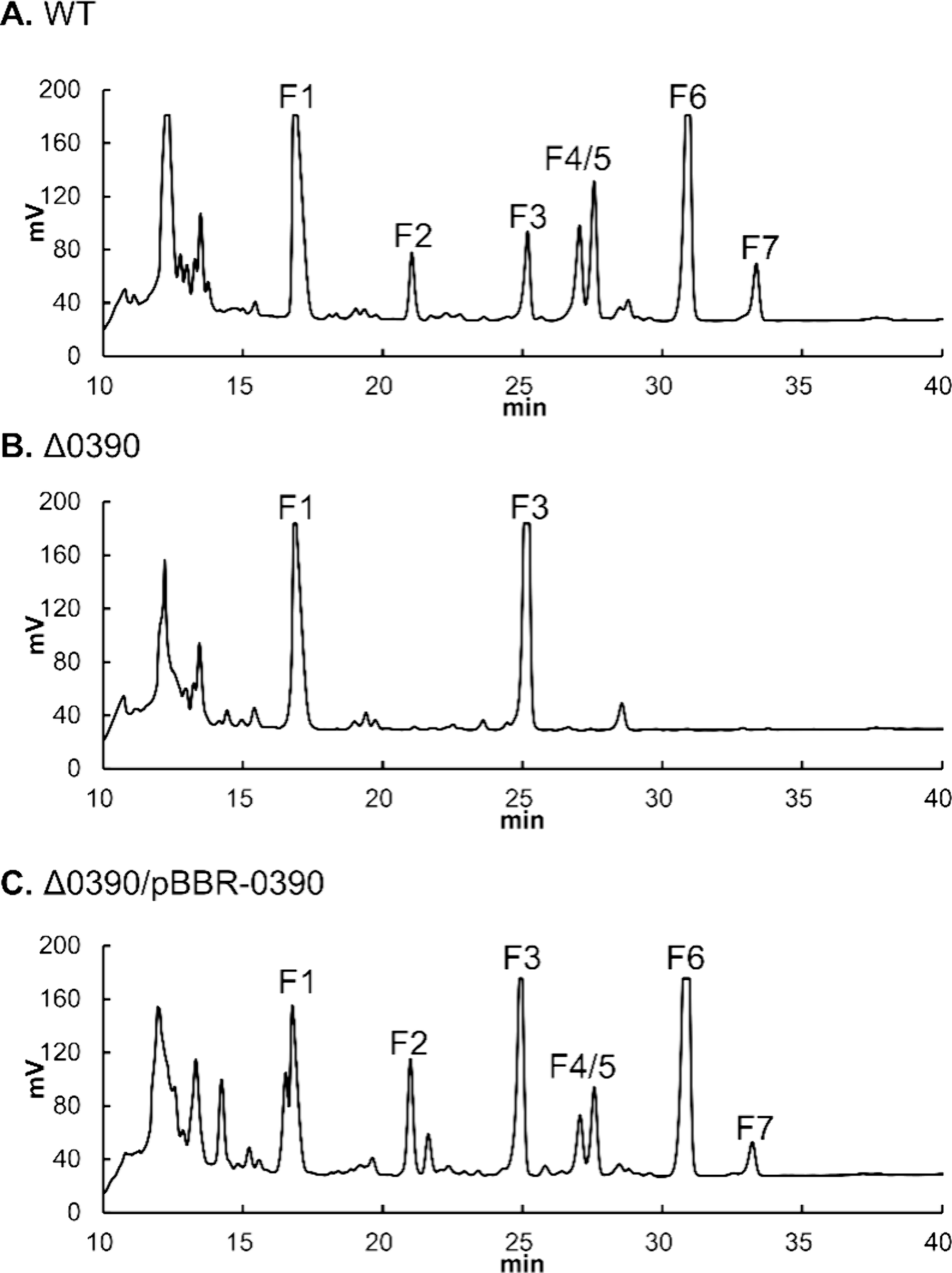
RP-HPLC chromatograms of secondary metabolites produced by *B. gladioli* BBB-01. Metabolites produced by WT (A), Δ0390 (B), and the complemented strain (Δ0390/pBBR-0390) (C) were extracted from cultivated agar and analyzed by RP-HPLC.

In addition to these purified peaks, antiSMASH also predicted high-confidence BGCs most similar to sinapigladioside (region 1.3), caryoynencin (region 1.4), plantaribactin (region 2.3), and an icosalide A/B-like cluster (region 2.12) (Table S3). The two NRPS BGCs in regions 2.4 and 2.9 were manually analyzed using BLASTN, which supports their assignment to haereogladiodin and burriogladiodin, respectively (Table S3). LC–MS analysis of crude extracts revealed putative features consistent with reported ions for several of these predicted products, including a feature at RT ∼ 12.7 min detected only in ESI– (*m*/*z* ∼ 468.2; Figure S3A), matching the reported ion(s) of sinapigladioside;^13^ a low-abundance feature at RT ∼ 36.5 min in ESI+ (*m*/*z* ∼ 1105.8; Figure S3B) that was not detected in PDA/UV chromatograms at 220 or 254 nm, matching reported masses for plantaribactin/gladiobactin;^35^ and a feature at RT ∼ 51 min in ESI+ (*m*/*z* ∼ 713.9; Figure S3C), matching the reported [M + H]+ of icosalide A1.^11^ In contrast, EICs targeting the expected *m*/*z* values for caryoynencin did not reveal detectable signals under our extraction and LC–MS conditions. Similarly, no signals were detected when targeting expected *m*/*z* values for multiple reported burriogladiodins and haereogladiodins, consistent with prior reports that these pathways can remain silent under standard laboratory culture conditions.^36^ The structures of purified compounds (F3–F7) were further elucidated by ^1^H, ^13^C, and 2D NMR spectroscopy. Key COSY and HMBC correlations supporting the planar structures of compounds F3–F7 are summarized in Figure S4.

Compound F3 was identified as gladiostatin, based on comprehensive analysis of its ^1^H and ^13^C NMR spectra, 2D NMR correlations (COSY, HSQC, HMBC, and NOESY; Table S4 and Figures S5–S10), and HRMS (*m*/*z* 506.2758 [M + H]^+^), in agreement with previously reported data.^12,15,37^ COSY and HMBC correlations established the planar connectivity of the glutarimide moiety, the polyketide chain, and the terminal butenolide unit, while NOESY correlations among H-9, H-10, and the 8-methyl group were consistent with the reported geometry of the C9=C10 double bond in gladiostatin (Figure S11A–C). In addition, a degradation product of gladiostatin was identified (Table S5 and Figures S12–S16). Its diagnostic NMR features are consistent with compound 5 reported by Chen et al.^37^ We propose that the degradation arises from the transformation of the butenolide moiety, involving steps of hydration-induced ring opening followed by decarboxylation and subsequent rearrangement, yielding the observed degradation product (Figure S17).

The antimicrobial activity of gladiostatin was assessed by determining its MIC against several opportunistic pathogens (Table 1). Gladiostatin exhibited promising activity against *C. glabrata*, with a MIC value of 8 μg/mL, followed by *C. parapsilosis*. In contrast, its inhibitory activity against *C. albicans* and the tested bacteria was relatively weak. This antagonistic property is comparable to the previously characterized attributes of gladiofungin/gladiostatin.^12,15^

**Table 1:** Antimicrobial Activity of Gladiostatin

tested microorganisms	MIC (μg/mL)
*S. aureus*	>64
*K. pneumoniae*	>64
*P. aeruginosa*	>64
*C. albicans*	>64
*C. glabrata*	8
*C. parapsilosis*	64

Compounds F6 and F7 were identified as gladiolin and isogladiolin, respectively (Figure 3), via a comparison of their NMR spectra (Figures S18–S21) and LC-MS with those previously reported.^14^ Consistent with the previous report, both compounds have the same molecular weight; however, isogladiolin elutes slightly later than gladiolin on a C18 column. Notably, the same study has shown that gladiolin, when dissolved in methanol and left at room temperature, can progressively convert to isogladiolin.

**Figure 3:**
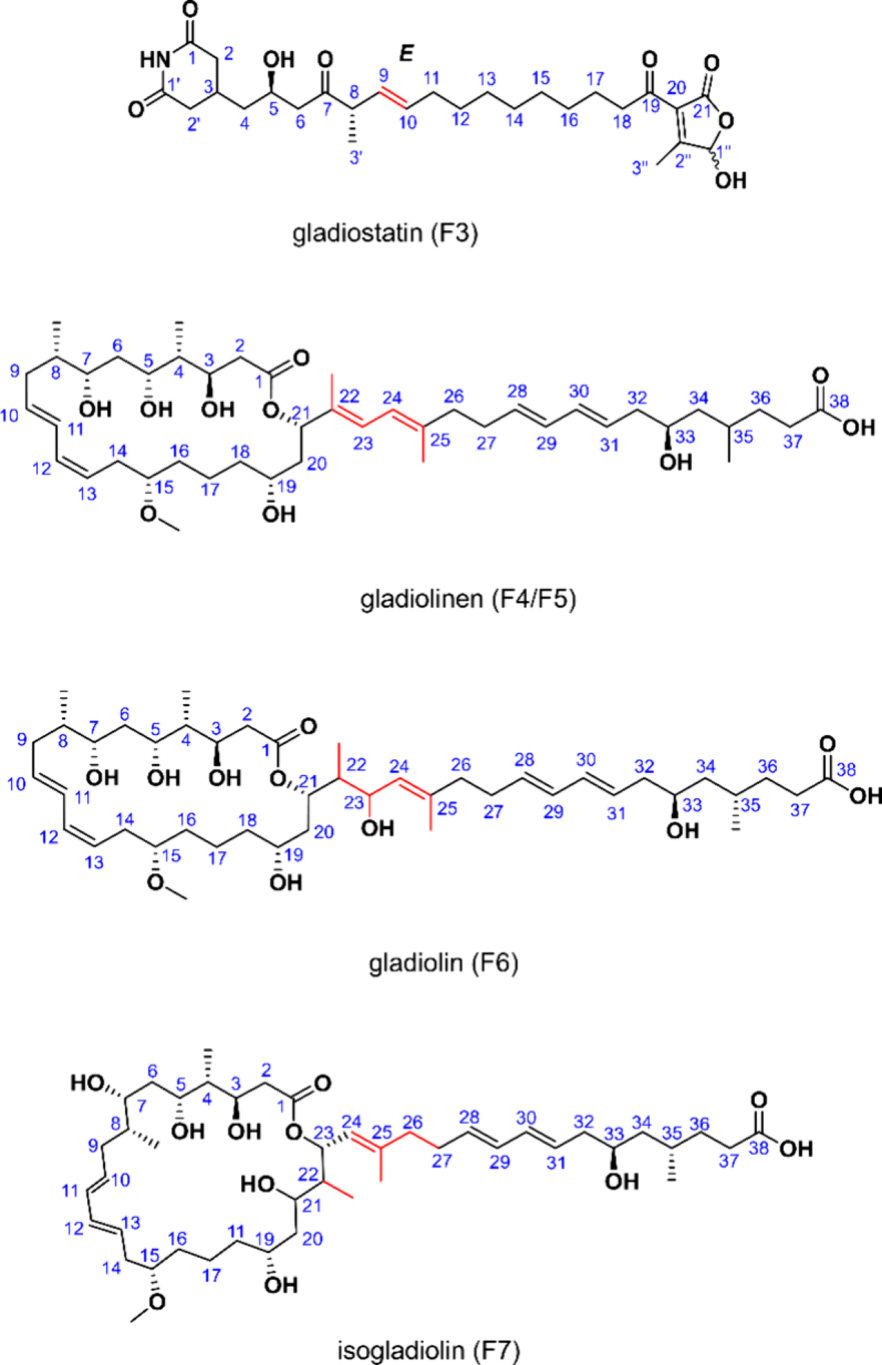
Chemical structures of gladiostatin (F3), gladiolinen (F4/F5), gladiolin (F6), and isogladiolin (F7).

As for fractions F4 and F5, no differences were observed in their ^1^H NMR spectra (Figures S22, S23), and both fractions exhibited identical molecular weights. Consequently, F4 and F5 were combined for detailed 2D NMR analysis. Structural elucidation based on ^1^H, ^13^C, COSY, TOCSY, HSQC, HMBC, and NOESY experiments (Table S6 and Figures S24–S30) demonstrated that the compound present in both fractions corresponds to a dehydrated derivative of gladiolin, resulting from the loss of a water molecule. Therefore, we named this compound gladiolinen (Figure 3). Key NOESY correlations supporting the relative configuration are shown in Figure S31. NOESY analysis of the combined F4/F5 sample revealed a single set of diagnostic NOE correlations, specifically between H-7 and the 8-methyl group, with no observable NOE between H-7 and H-8 (Figure S30), indicating a single dominant relative configuration. LC–MS analysis using a C18 column revealed two closely eluting chromatographic features within the F4/F5 fraction (Figure 2); however, these features could not be distinguished by NMR spectroscopy or chiral LC–MS (Figure S32), indicating that the observed separation does not necessarily reflect distinct chemical entities. The absolute configuration of gladiolinen remains to be established by future chiroptical analyses. The compounds of F1 and F2 have not been successfully identified, primarily because they were unstable during the purification process.

### Effects of lttr_0390 Disruption on Antagonism

To investigate the multifaceted roles of LTTRs in *B. gladioli* BBB-01 under environmental stimuli, we performed a BLASTp analysis to identify the LTTR most closely resembling ScmR in terms of amino acid sequence. This analysis pinpointed LTTR_0390 as the closest match from a pool of over 200 putative LTTRs, making it a candidate for further mutagenesis studies.

LTTR_0390 comprises 326 amino acid residues (Figure S33) and shares 65.9% sequence identity with ScmR. The presence of a putative lux box in the promoter region suggests that the expression of LTTR_0390 is regulated by QS. To investigate its function, we generated the knockout strain Δ0390, and the targeted recombination was validated by PCR (Figure S34). To test whether the altered antagonism was attributable to *lttr_0390* disruption, antagonistic activity of the WT, Δ0390, and the complemented strain Δ0390/pBBR-0390 was evaluated using double-layer and confrontation assays. Overall, bacterial antagonistic activity was markedly reduced in Δ0390, whereas antifungal activity was enhanced; for example, inhibition of *S. aureus* was barely detectable, while inhibition of *C. albicans* and *M. oryzae* was increased (Figure 4A,B). The complemented strain Δ0390/pBBR-0390 restored antagonism against *S. aureus*; in contrast, it reduced the enhanced antifungal phenotype toward WT levels for *C. albicans* and *M. oryzae* (Figure 4A,B), indicating that these activity shifts are attributable to *lttr_0390* disruption. To connect the altered antagonistic phenotypes with metabolite output, we performed LC–MS EIC-based semiquantitative analysis of crude extracts from WT and Δ0390 (Figure S35). Gladiostatin (F3) was significantly increased in Δ0390 relative to WT (2.08-fold by mean EIC area; *p* < 0.001), corresponding to an increase from 142.3 ± 7.0 to 295.9 ± 11.3 μg of gladiostatin per mg crude extract (Figures S35A,C). In contrast, the gladiolin/isogladiolin (F6+F7) associated feature was only detected in WT but not in Δ0390 (Figures S35B,C). Since gladiolin and gladiostatin predominantly inhibit Gram-positive bacteria and fungi, respectively, this alteration in metabolite production upon *lttr_0390* disruption is consistent with the observed changes in antimicrobial activity.

**Figure 4:**
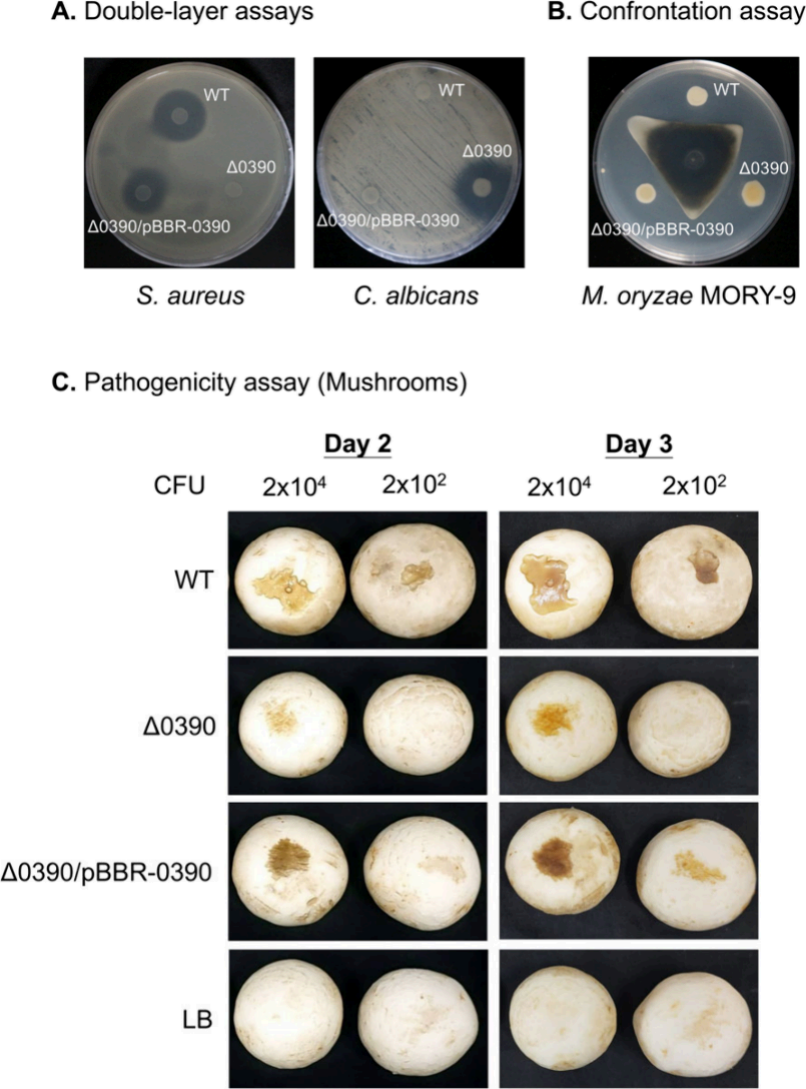
Effects of *lttr_0390* disruption on the bacterial antagonistic activity and pathogenicity. (A) Antagonistic activity against *S. aureus* and *C. albicans*. (B) Antagonistic activity against *M. oryzae*. (C) Pathogenicity toward the mushroom *A. bisporus*. The bacterial colony-forming unit (CFU) applied to each mushroom cap is indicated. LB medium was administered in the negative control group.

Because the BBB-01 strain belongs to pv *agaricicola*, we evaluated whether disruption of *lttr_0390* affects pathogenicity toward *A. bisporus*. The WT strain caused prominent lesions and tissue maceration on the mushroom surface on day 3 (Figure 4C). In contrast, the Δ0390 strain exhibited markedly attenuated pathogenicity, resulting in substantially smaller and less severe lesions. The complemented strain restored pathogenicity, despite not reaching the level of the WT, indicating that the loss of virulence in Δ0390 is attributable to disruption of *lttr_0390*.

### Changes in Secondary Metabolite Production Profile

Production of EA-extractable secondary metabolites was analyzed by RP-HPLC. Disruption of *lttr_0390* led to a marked reduction of peaks corresponding to gladiolinen (F4/F5), gladiolin (F6), and isogladiolin (F7), while the intensity of the gladiostatin peak (F3) was markedly increased (Figure 2B). The peak corresponding to F2, an unidentified compound, was also substantially reduced in the mutant strain. The complemented strain largely restored the WT secondary metabolite profile, including reappearance of the missing peaks and a reduced level of gladiostatin (Figure 2C). These results indicate that LTTR_0390 is essential for the balanced biosynthesis of multiple bioactive metabolites.

Besides water-diffusible secondary metabolites, *B. gladioli* BBB-01 emits VOCs to counteract competitors or communicate with surrounding organisms. VOCs emitted by WT and Δ0390 strains were analyzed by using solid-phase microextraction followed by GC-MS analysis. Disruption of the *lttr_0390* significantly reduced the emission levels of sulfur-containing VOCs, including DMDS and SMT (Figure 5A,B).

**Figure 5:**
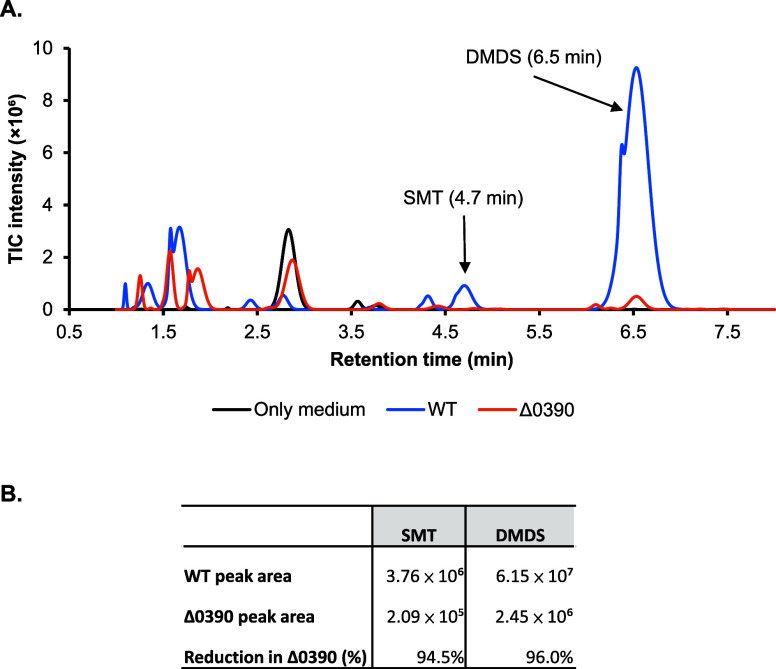
Changes in volatilome profiles upon disruption of *lttr_0390*. (A) Total ion chromatograms (TICs) of volatiles from the growth medium control (Only medium), WT, and Δ0390. Peaks corresponding to SMT and DMDS are indicated by arrows. (B) Integrated peak areas for SMT and DMDS from the chromatograms shown in (A).

### Effects of *lttr_0390* Disruption on QS

Bacteria utilize QS to regulate specific phenotypic traits, such as biofilm formation and motility, in a population density-dependent manner. To investigate the role of *lttr_0390* in the QS circuitry, the production of QS autoinducers was compared between the WT and Δ0390 strains, while biofilm formation and bacterial motility were assessed across the WT, Δ0390, and Δ0390/pBBR-0390.

The *Chromobacterium violaceum* CV026 biosensor strain has been employed to detect the presence of autoinducers produced by the test strains.^38^ This biosensor produces a purple pigment in response to the presence of N-acyl homoserine lactones (AHLs), such as *N*-hexanoyl homoserine lactone (C6-HSL) or *N*-octanoyl homoserine lactone (C8-HSL). Both WT and Δ0390 produced the necessary autoinducers (Figure 6A), indicating that *lttr_0390* disruption did not impair AHL production.

**Figure 6:**
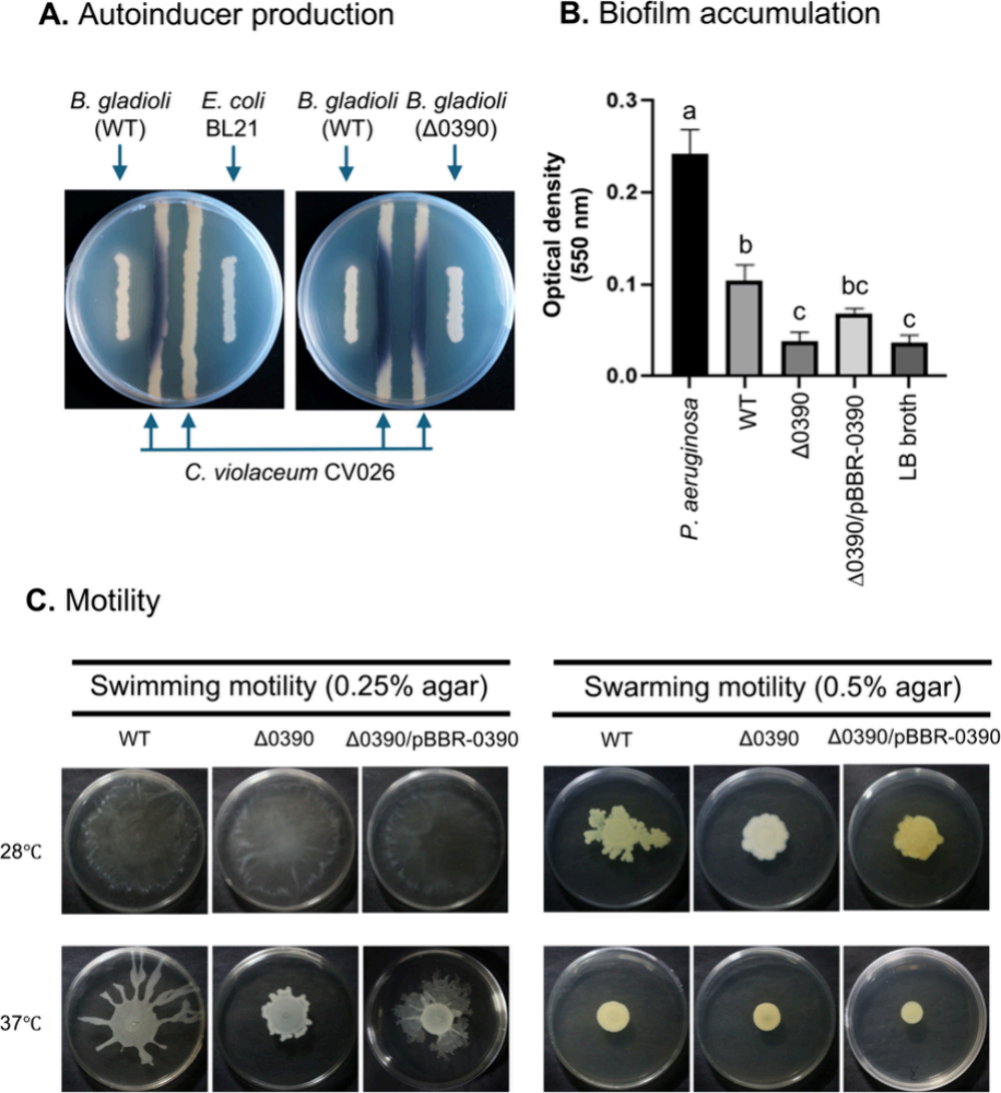
Effects of *lttr_0390* disruption on quorum-sensing-related phenomenon. (A) Production of AHL autoinducers by the WT and Δ0390 strains was tested using the biosensor strain *C. violaceum* CV026. (B) Biofilm formation by the indicated strains in LB medium was quantified by crystal violet staining. Statistical significance was assessed by one-way ANOVA with Tukey’s multiple comparisons test. Different letters above bars indicate significant differences (*p* < 0.05). (C) Motility of WT, Δ0390, and the complemented strain (Δ0390/pBBR-0390) on LB agar plates containing 0.25% or 0.5% agar.

The ability of *B. gladioli* strains to form biofilms was quantified using the crystal violet staining assay. Disruption of *lttr_0390* moderately reduced biofilm formation compared with the WT, indicating that this regulator plays a role in biofilm development. The complemented strain partially restored biofilm production (Figure 6B). The swimming and swarming motility were evaluated on 0.25 and 0.5% (w/v) agar plates, respectively. Motility of the Δ0390 was markedly reduced compared with the WT (Figure 6C). These effects were particularly pronounced at 37 °C for swimming motility and at 28 °C for swarming motility. The complemented strain Δ0390/pBBR-0390 restored the motility, particularly swimming motility at 37 °C (Figure 6C).

### Transcriptomic Analysis

Transcriptomic changes resulting from *lttr_0390* disruption were analyzed by RNA sequencing (RNA-seq). In the WT sample, the value of transcript per million for *lttr_0390* was 121, compared to 1010 of the housekeeping control, ribosomal protein L32. Differentially expressed genes (DEGs) between the Δ0390 and WT strains, both grown on PDA, are presented in the volcano plot (Figure 7). DEGs were classified as upregulated if they had a fold change >2 and a *p*-value <0.05, or downregulated if their fold change was <0.5 with a *p*-value <0.05. Among the 224 upregulated genes, 40 exhibited differential expression with FDR-adjusted *p*-value (*q*-values) <0.05, while 47 out of the 147 downregulated genes met the same statistical threshold.

**Figure 7:**
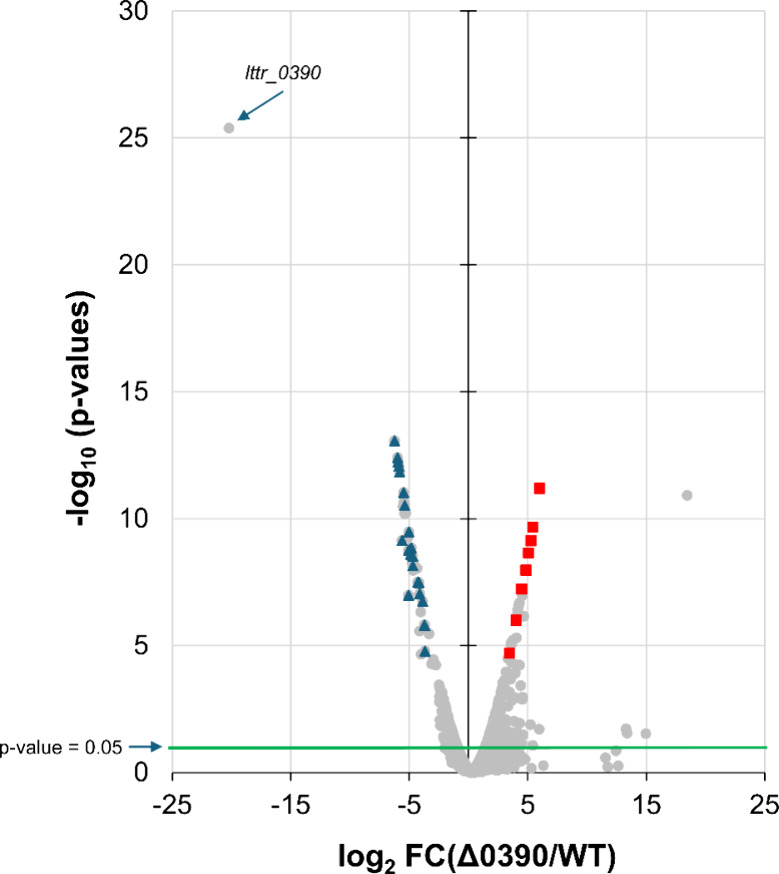
RNA-seq volcano plot showing differentially expressed genes between WT and Δ0390 strains. Red squares indicate BGC genes involved in gladiostatin biosynthesis, while blue triangles indicate genes associated with gladiolin biosynthesis.

### Upregulated BGCs upon *lttr_0390* Disruption

A significant proportion of the upregulated genes are linked to the predicted BGCs in regions 2.1 and 2.4, respectively, which are responsible for synthesizing gladiostatin and haereogladiodin (Table S3). The gene organization of these two BGCs, along with the fold change for each key gene, is shown in Figure 8A,B. Detailed information, including protein ID, gene description, locus location, and others, is provided in Tables S7 and S8. Consistent with increased transcript abundance from the gladiostatin BGC, gladiostatin levels were significantly elevated, as quantified by EIC-based semiquantitative LC–MS analysis (Figure S35). Although region 2.4 (haereogladiodin BGC) was significantly upregulated in Δ0390, haereogladiodins were not detected under our current extraction and LC–MS/HPLC conditions. The transcriptomic analysis also uncovered a potential BGC based on the coupregulation of its associated genes (Figure 8C and Table S9), although it was not predicted by antiSMASH. This BGC, characterized by an *N*-acyl amino acid synthase, is hypothesized to produce *N*-acyl amino acid derivatives that may function as signaling molecules, antimicrobials, or siderophores.

**Figure 8:**
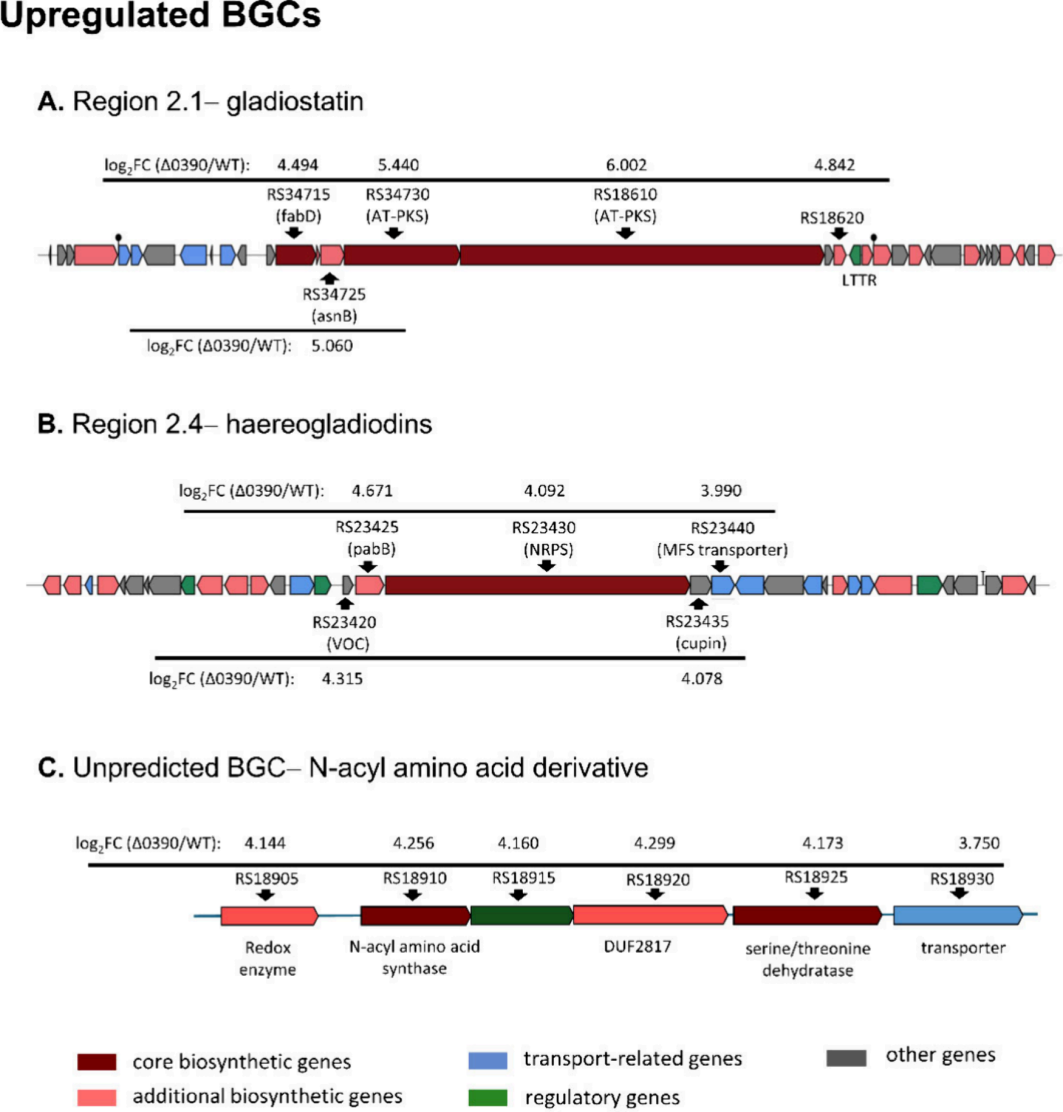
Upregulated BGCs upon disruption of lttr_0390. The gladiostatin BGC (A) was predicted by antiSMASH. The haereogladiodin NRPS locus (B) is supported by comparative analysis against reference BGC sequences and by RNA-seq upregulation in Δ0390. A putative gene cluster for *N*-acyl amino acid derivatives (C) is proposed based on RNA-seq coupregulation. Fold changes are shown as log_2_FC (Δ0390/WT). Gene names in the format JKG63_RSxxxxx are abbreviated as RSxxxxx (x: numbers).

Additionally, a small gene cluster encoding the components of cytochrome *bd* quinol oxidase (Table S10) was activated following *lttr_0390* disruption. Cytochrome *bd* catalyzes oxygen reduction to water by utilizing quinol as the electron donor; besides, it exhibits quinol peroxidase activity.^39^ The physiological significance of cytochrome *bd* upregulation in the Δ0390 strain remains uncertain.

To ascertain whether the upregulation of the BGCs for gladiostatin, haereogladiodins, and the putative N-acyl amino acid derivative was truly dependent on *lttr_0390* disruption, a comparative transcriptomic analysis between the Δ0390 strain and the complemented strain Δ0390/pBBR-0390 was performed. The RNA-seq data showed that reintroduction of *lttr_0390* into the Δ0390 strain indeed brought down the expression levels of these BGCs (Tables S7–S10).

### Downregulated BGCs upon *lttr_0390* Disruption

The disruption of *lttr_0390* led to a significant suppression of BGCs in regions 1.3 and 2.10, responsible for the production of sinapigladioside and gladiolin, respectively (Figure 9A,B). Detailed information, including protein IDs, gene descriptions, and locus locations of the associated genes, is provided in Tables S11 and S12. Downregulation of the gladiolin BGC is accompanied by a marked reduction or complete loss of gladiolin, isogladiolin, and gladiolinen production, as demonstrated by EIC-based semiquantitative LC–MS analysis and consistent with the HPLC profile (Figure 2; Figure S35). The antiSMASH server predicted an icosalide-like BGC in region 2.12. In Δ0390, transcript levels for the NRPS gene JKG63_RS31505 within this region decreased to 5% of the WT level (Figure 9C), whereas other genes in the region were not significantly altered (Table S13). To verify whether the downregulation of the sinapigladioside and gladiolin BGCs, as well as the NRPS in icosalide BGC, is directly attributable to *lttr_0390* disruption, we compared the transcriptomes of the Δ0390 and the complemented strain Δ0390/pBBR-0390. RNA-seq analysis revealed that reintroducing *lttr_0390* into the Δ0390 background rebooted the expression levels of these BGCs (Tables S11–S13).

**Figure 9:**
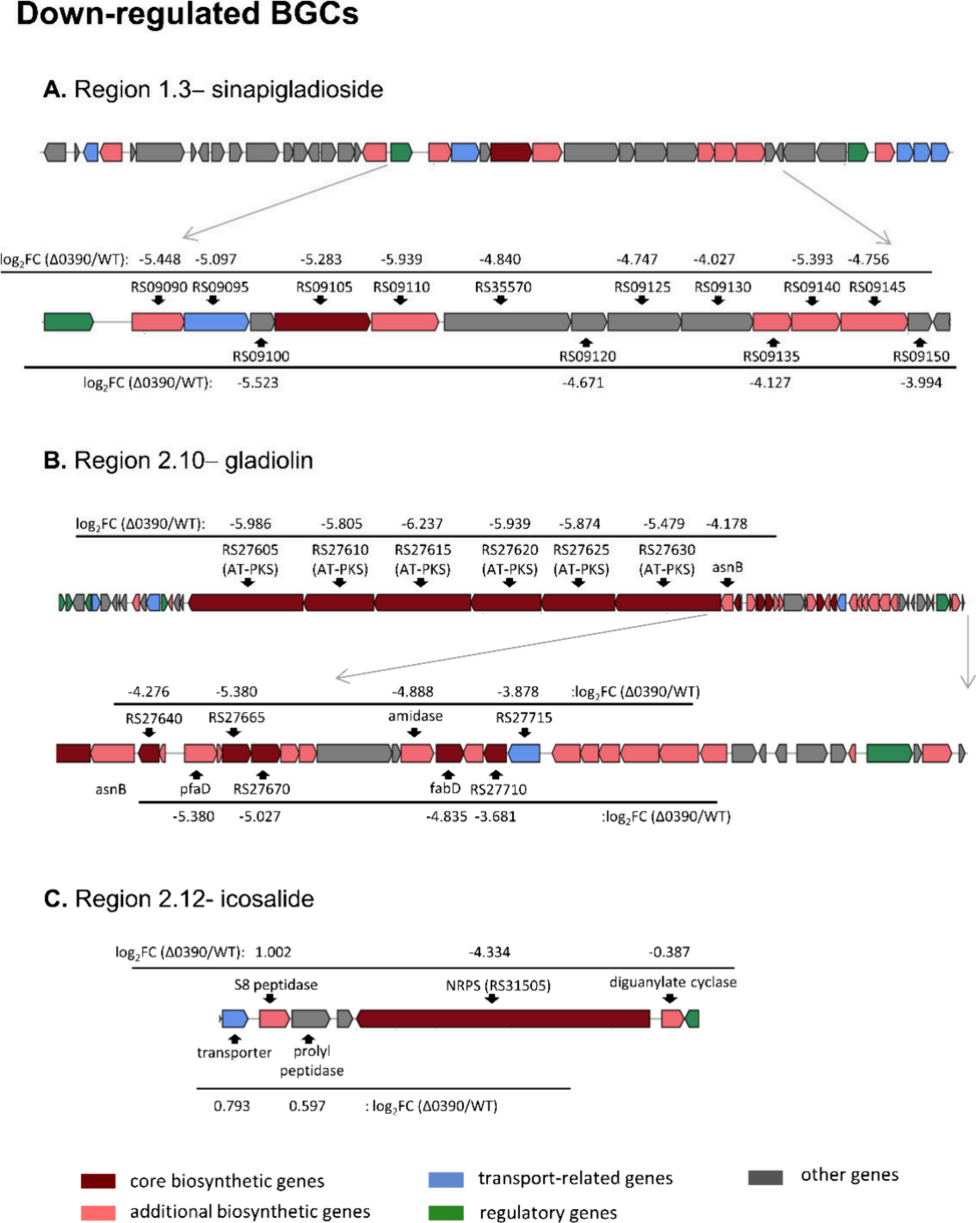
Downregulated BGCs upon the disruption of *lttr_0390*. BGCs for sinapigladioside (A), gladiolin (B), and icosalide (C) were predicted by antiSMASH. Fold changes are shown as log_2_FC (Δ0390/WT). Gene names in the format JKG63_RSxxxxx are abbreviated as RSxxxxx (x: numbers).

### Downregulation of Motility- And Biofilm Formation-Related Genes

Transcriptomic analysis revealed that the expression levels of the flagellar motor proteins MotA and MotB were moderately reduced following *lttr_0390* disruption (Table S14), which aligns with the observed motility decrease in Δ0390. The expression of fimbrial and pilus-associated proteins, which are essential for biofilm formation, was also moderately reduced (Table S14), correlating with the diminished biofilm production observed in Δ0390.

## Discussion

Consistent with the metabolic versatility reported across *Burkholderia* spp.^40^ and with prior reports on *B. gladioli* strains,^11–14^ *B. gladioli* BBB-01 produces a diverse array of secondary metabolites. NMR analyses following EA extraction and RP-HPLC purification identified gladiolin, gladiolinen, isogladiolin, and gladiostatin. Comparative transcriptomic analysis of the WT and Δ0390 strains suggests additional biosynthetic capacity in BBB-01, including sinapigladioside, icosalide, and a putative *N*-acyl amino acid derivative pathway. BLASTN analysis further indicates that BBB-01 harbors haereogladiodin and burriogladiodin biosynthetic loci (Table S3). The unresolved RP-HPLC peaks F1 and F2 further highlight the strain’s rich secondary metabolite repertoire.

QS enables bacteria to coordinate density-dependent traits, including secondary metabolism, motility, biofilm formation, and virulence. The primary QS system in *B. gladioli* is governed by *LuxIR* homologues.^41^ The *LuxI* homologue encodes an AHL synthase, primarily producing C8-HSL, followed by C6-HSL.^42^ *LuxR* homologues function as transcriptional regulators that bind AHL autoinducers and activate QS-dependent gene expression, thereby regulating various physiological processes. Inactivation of the AHL synthase gene in *B. gladioli* ICMP11096 resulted in reduced antimicrobial activity, decreased secretion of protease and chitinase, and diminished damage to the pseudotissues of *A. bisporus*.^8^ Although the disruption of *lttr_0390* in the BBB-01 strain does not affect the production of acyl-homoserine lactones, it negatively affects some QS-dependent phenotypes, such as bacterial motility and biofilm formation, likely by downregulating the production of flagella motor proteins, pilus, and fimbrial proteins.

Temperature-dependent control of motility has been reported in *Burkholderia* and can differentially affect swimming and swarming behaviors. In *Burkholderia glumae*, QS mutants are largely aflagellate at 37 °C and consequently lack swimming motility, whereas at 28 °C these mutants regain flagella and show increased expression of flagellar genes (e.g., *fliC* and *flgK*), indicating that QS control of flagellar biogenesis is temperature dependent.^43^ Swarming can also be constrained by QS-dependent production of a surface-wetting factor; in *B. glumae*, exogenous rhamnolipids restore swarming at 28 °C but not at 37 °C, consistent with flagellar availability becoming the dominant constraint at higher temperature.^44^ In *B. gladioli*, QS and an LTTR have been linked to swarming-associated phenotypes. The spreading of *B. gladioli* UAPS07070 on soft agar was greater at 30 °C than at 37 °C, and disruption of an LTTR gene (homologous to *lttr_1514* in *B. gladioli* BBB-01) reduced antagonism, toxoflavin production, motility, and virulence, underscoring temperature as an important variable and LTTRs as central regulators of QS-linked outputs in this species.^29^ Importantly, our transcriptomic profiling was conducted at 28 °C and supports the phenotypes observed at 28 °C, as *lttr_0390* disruption reduced expression of the flagellar motor genes *motA/motB* and multiple fimbrial/pilus-associated genes (Table S14). Together, these precedents suggest that the greater phenotypic changes we observed at 37 °C for swimming and at 28 °C for swarming reflect distinct temperature-sensitive limiting steps (flagellar biogenesis/function at higher temperature versus surface-associated wetting/surfactant availability at lower temperature), potentially coordinated through LTTR_0390-dependent regulatory reprogramming.

Many LTTRs function as dual repressor-activators, enabling bacteria to fine-tune their metabolism and stress responses. A single LTTR with this characteristic can activate some operons or genes while repressing others depending on the context of the target gene, promoter architecture, and the presence of cofactors or small-molecule inducers.^21,22^ The LTTRs ScmR and PhcA represent notable examples. In *B. thailandensis* E264, ScmR regulates quorum sensing, pH homeostasis, virulence, and secondary metabolism, including suppression of multiple BGCs (e.g., malleilactone, capistruin, thailandamide, bactobolin, burkholdac, and malleobactin) directly or indirectly via cluster-specific regulators, while stimulating the BGC for 4-hydroxy-3-methyl-2-alkylquinoline.^23,45^ The LTTR PhcA in *R. solanacearum* globally regulates a large fraction of the genome when the bacterium is cultivated in complete medium and modulates numerous transcription factors,^46^ illustrating a hierarchical architecture with both direct and indirect regulatory control.

Consistent with this dual activator-repressor paradigm, the *B. gladioli* BBB-01 genome is predicted to harbor more than 200 LTTR-encoding genes. The present study demonstrated the diverse functions of LTTR_0390 in *B. gladioli* BBB-01 (Figure 10). LTTR_0390 is required to activate BGCs located in chromosomal regions 1.3, 2.10, and 2.12, promoting the synthesis of sinapigladioside, gladiolin, and icosalide. On the other hand, it negatively controls BGCs in regions 2.1 and 2.4, along with an unpredicted BGC, potentially setting up production thresholds for gladiostatin, haereogladiodins, and an unknown *N*-acyl amino acid derivative, respectively. LTTR_0390 is also required for the emission of DMDS and SMT. Modulating the production profile of secondary metabolites may enhance the competitive advantage of the BBB-01 strain, allowing it to outcompete diverse surrounding organisms across varying growth conditions. Additionally, LTTR_0390 appears to negatively regulate the expression of cytochrome *bd* quinol oxidases. Overall, LTTR_0390 acts as a key executor in the QS circuitry by orchestrating the antibiotic production profile and controlling many QS-dependent behaviors in *B. gladioli* BBB-01.

**Figure 10:**
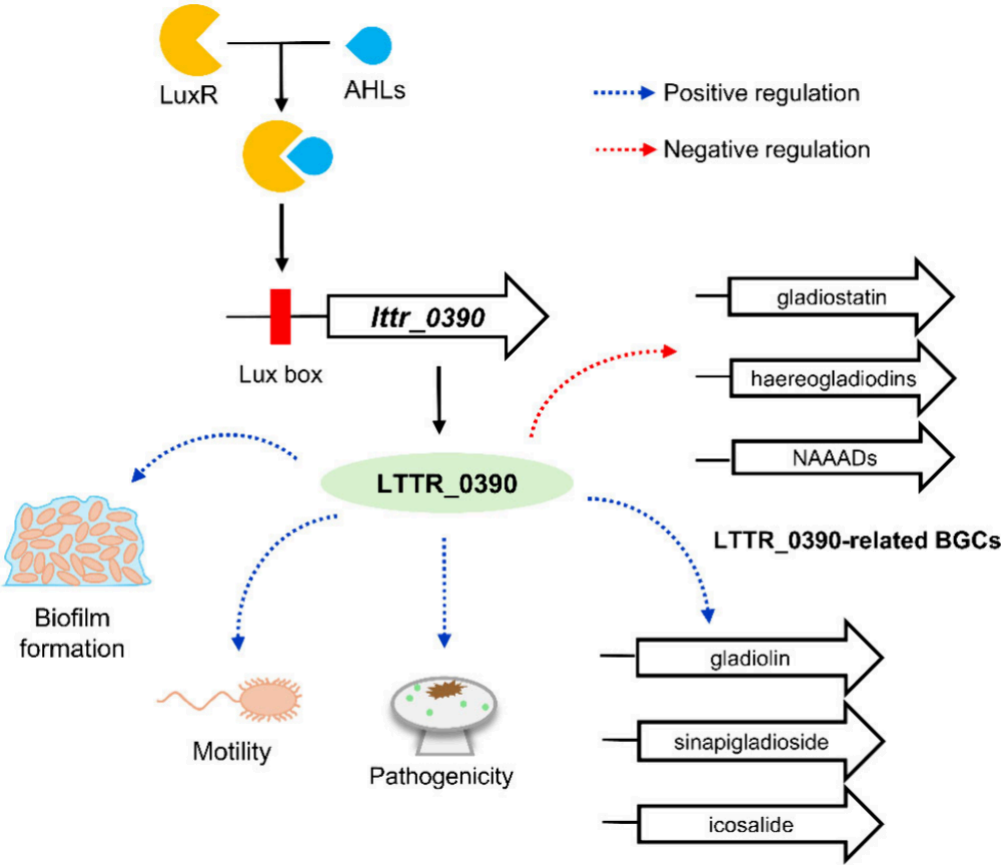
Proposed regulatory role of LTTR_0390 in *B. gladioli* BBB-01. *lttr_0390* is controlled by a lux box located upstream of the gene, influenced by the binding of the LuxR/AHLs complex. LTTR_0390 plays a crucial role in various cellular functions associated with quorum sensing, including biofilm formation, motility, pathogenicity, and the production of secondary metabolites with antibiotic and antifungal properties. NAADs: *N*-acyl amino acid derivatives.

Soft rot disease of *A. bisporus* caused by *B. gladioli* pv *agaricicola* leads to substantial economic losses in the mushroom industry. The pathogenicity of this bacterium toward *Agaricus* has been attributed to the production of extracellular enzymes and toxins.^8^ In particular, several secondary metabolites, including toxoflavin, caryoynencin, and sinapigladioside, have been detected during the infection of edible mushrooms by *B. gladioli* pv *agaricicola* HKI0676.^9^ In the present study, disruption of *lttr_0390* significantly reduced transcription of the sinapigladioside BGC, suggesting sinapigladioside’s role as a key virulence factor in soft rot disease. The impaired motility observed in the Δ0390 strain is likely to further contribute to its attenuated pathogenicity. Strikingly, while the production of gladiostatin, an antifungal metabolite against *Candida* spp., was remarkably enhanced due to *lttr_0390* disruption, tissue damage to mushrooms was diminished, uncoupling antifungal capacity from mushroom injury. Overall, soft rot in *A. bisporus* appears to be a multifactorial disease. Further investigations into the regulatory functions of LTTRs may uncover additional virulence factors relevant to this economically important pathogen.

This study demonstrated that LTTR_0390 functions both as a transcriptional activator and repressor in *B. gladioli* BBB-01. LTTR_0390 likely binds to distinct operator sites within the promoters of its target BGCs, with the regulatory outcome, activation or repression, being influenced by local DNA topology, the presence of specific effectors, or interactions with additional pathway-specific transcription factors. The molecular basis of these opposing regulatory outcomes remains to be determined. Transcriptomic analysis in this study also revealed that LTTR_0390 upregulates the expression of a LacI family DNA-binding transcriptional regulator and a CysB family HTH-type regulator, while downregulating a LuxR C-terminal domain-containing transcriptional regulator. How these downstream regulators integrate with LTTR_0390 to shape BGC expression and QS-linked phenotypes warrants further investigation.

## Supplementary Material



## Data Availability

The genomic data supporting the findings of this study can be retrieved from the National Center for Biotechnology Information (NCBI) with the *B. gladioli* BBB-01 genome assembly number GCA_016698705.1.

## Notes

LC-MS data of F1 to F7; HRMS data of gladiostatin (F3) and gladiolin (F6); LC-MS detection of putative secondary-metabolite features; key COSY and HMBC correlations of F3–F7; 1H, 13C, COSY, HSQC, HMBC, and NOESY NMR data of F3 to F7; geometric assignment of gladiostatin (F3); proposed degradation mechanism for gladiostatin (F3); chiral LC-MS analysis of gladiolinen (F4/5); nucleotide sequence of the promoter and ORF; PCR confirmation of *lttr_0390* disruption; EIC-based comparison of F3 and F6/F7 in crude extracts; microorganisms used in this study; primer sequences; predicted secondary metabolite BGCs in the B. gladioli BBB-01 genome; fold changes of the transcripts in up- and downregulated BGCs upon *lttr_0390* disruption; fold changes of transcripts related to motility and biofilm formation (PDF)

## Abbreviations

AHL(s): *N*-acyl homoserine lactone(s)
antiSMASH: antibiotics and secondary metabolite analysis shell
BGC(s): biosynthetic gene cluster(s)
CAR/PDMS: carboxen/polydimethylsiloxane
CFU: colony-forming unit
COSY: correlation spectroscopy
DEGs: differentially expressed genes
DMDS: dimethyl disulfide
EA: ethyl acetate
ESI (+): electrospray ionization (positive mode)
ESI (−): electrospray ionization (negative mode)
GC-MS: gas chromatography–mass spectrometry
HMBC: heteronuclear multiple-bond correlation
HSQC: heteronuclear single-quantum correlation
HSL: homoserine lactone
HPLC: high-performance liquid chromatography
LC-MS: liquid chromatography–mass spectrometry
LB: lysogeny broth (Luria–Bertani medium)
LTTR(s): LysR-type transcriptional regulator(s)
MIC: minimum inhibitory concentration
mRNA: mRNA
NB: nutrient broth
NIST20: National Institute of Standards and Technology 2020 mass spectral library
NMR: nuclear magnetic resonance
NOESY: nuclear Overhauser effect spectroscopy (NMR)
NRPS: nonribosomal peptide synthase
OD_260_ /OD_600_: optical density at 260 nm/600 nm
ORF: open reading frame
PCR: polymerase chain reaction
PDA: potato dextrose agar
pv: pathovar
QS: quorum sensing
RNA-seq: RNA sequencing
RP-HPLC: reverse-phase HPLC
SMT: *S*-methyl thioacetate
SPME: solid-phase microextraction
TOCSY: total correlation spectroscopy (NMR)
UV/vis: ultraviolet/visible
VOC(s): volatile organic compound(s)
WT: wild type
Δ0390: deletion mutant of gene *lttr_0390*
*m*/*z*: mass-to-charge ratio
