# Supplementary material for: The Crucial Role of LTTR_0390 in Burkholderia gladioli BBB-01 in Orchestrating Antibiotic Production, Quorum-Sensing Responses, and Pathogenicity on Mushrooms
Source: J Agric Food Chem. 2026 Mar 30;74(13):11271–84. doi: 10.1021/acs.jafc.5c12363 (PMC13067344; doi:10.1021/acs.jafc.5c12363)
Supplement: Supplementary file 1 [file jf5c12363_si_001.pdf]

Supplementary Information

The crucial role of LTTR_0390 in *Burkholderia gladioli* BBB-01 in orchestrating antibiotic production, quorum-sensing responses, and pathogenicity on mushrooms

Ali Diyapoglu[1,2,3,#], Yu-You Liu[1,#], Alican Abay[1], Kuan-Hung Lin[4], I-Wen Lo[5], Chi-Fon Chang[4], Yi-Ping Huang[4], Chi-Ting Chung[6], Tsung-Lin Li[4,7,*], Menghsiao Meng[1,*]

[1]Graduate Institute of Biotechnology, National Chung Hsing University, Taichung, 40227, Taiwan.

[2]Molecular and Biological Agricultural Sciences, Taiwan International Graduate Program, Academia Sinica and National Chung Hsing University, Taipei, 11529, Taiwan.

[3]Agricultural Biotechnology Research Center, Academia Sinica, Taipei, Taiwan.

[4]Genomics Research Center, Academia Sinica, Taipei, 11529, Taiwan.

[5]National Research Institute of Chinese Medicine, Ministry of Health and Welfare, Taipei, 112026, Taiwan.

[6]Bachelor Program in Biotechnology, National Chung Hsing University, Taichung, 40227, Taiwan.

[7]Biotechnology Center, National Chung Hsing University, Taichung, 40227, Taiwan.

*Corresponding authors.

E-mail addresses: mhmeng@dragon.nchu.edu.tw (M. Meng) and tlli@gate.sinica.edu.tw (T. L. Li)

#A.D. and Y.Y.L contributed equally to this work.

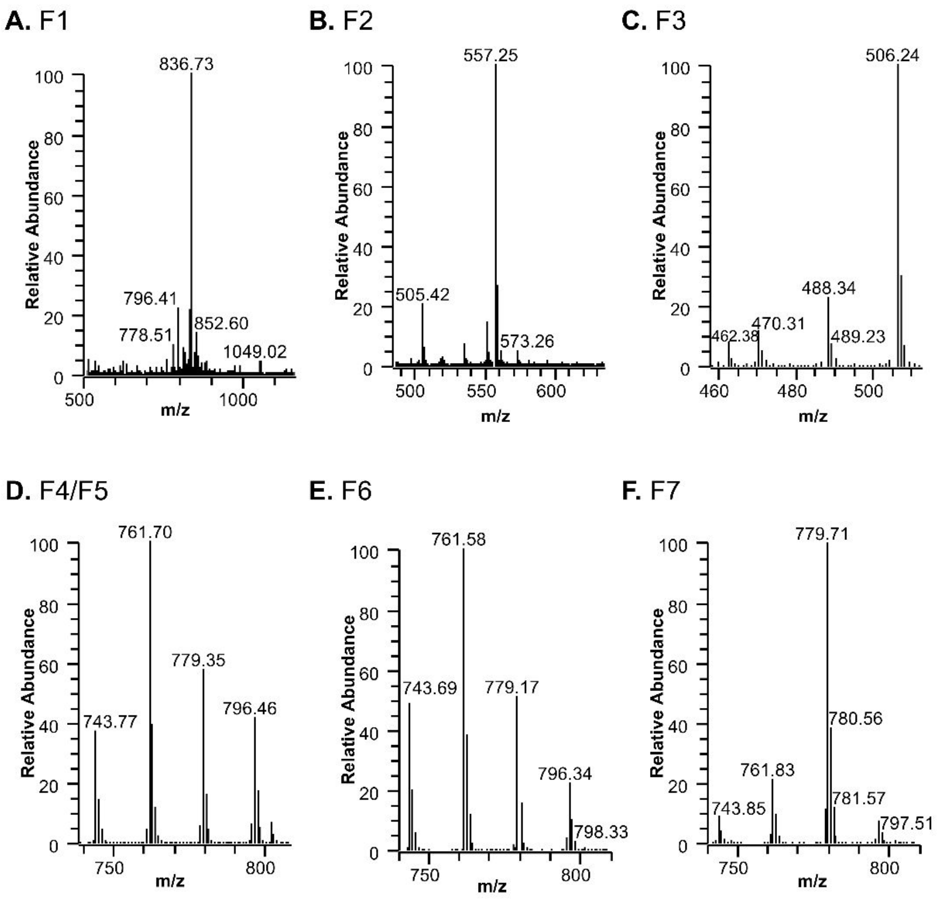

Figure S1. Mass spectral profiles of fractions F1–F7. Representative mass spectra (in ESI+ mode) are shown for **(A)** F1, **(B)** F2, **(C)** F3, **(D)** F4/F5, **(E)** F6, and **(F)** F7.

A. Gladiostatin (F3)

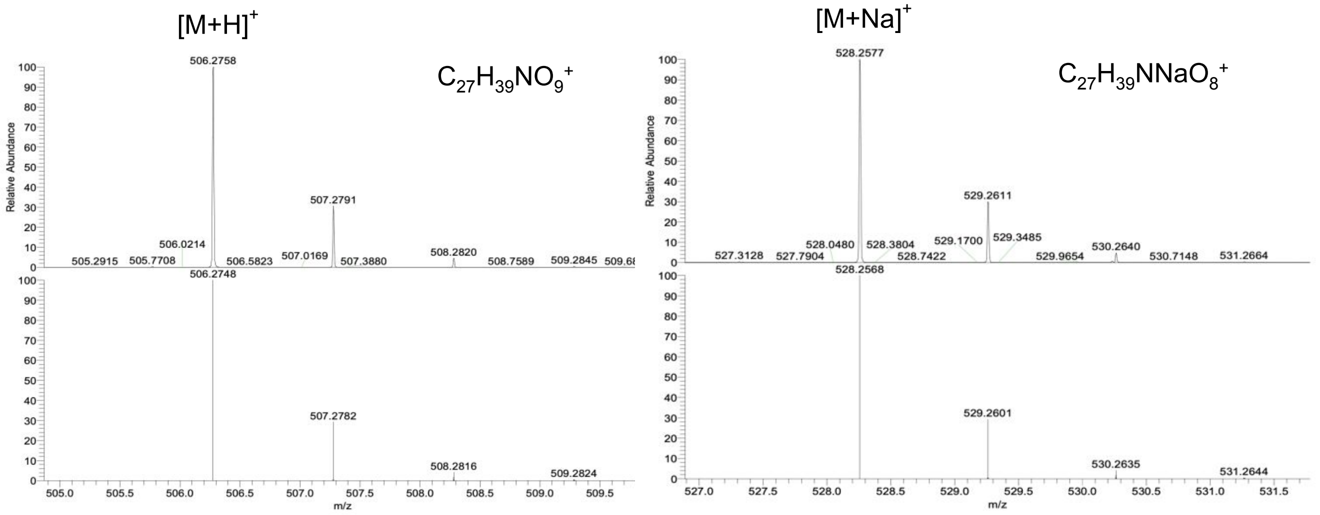

B. Gladiolin (F6)

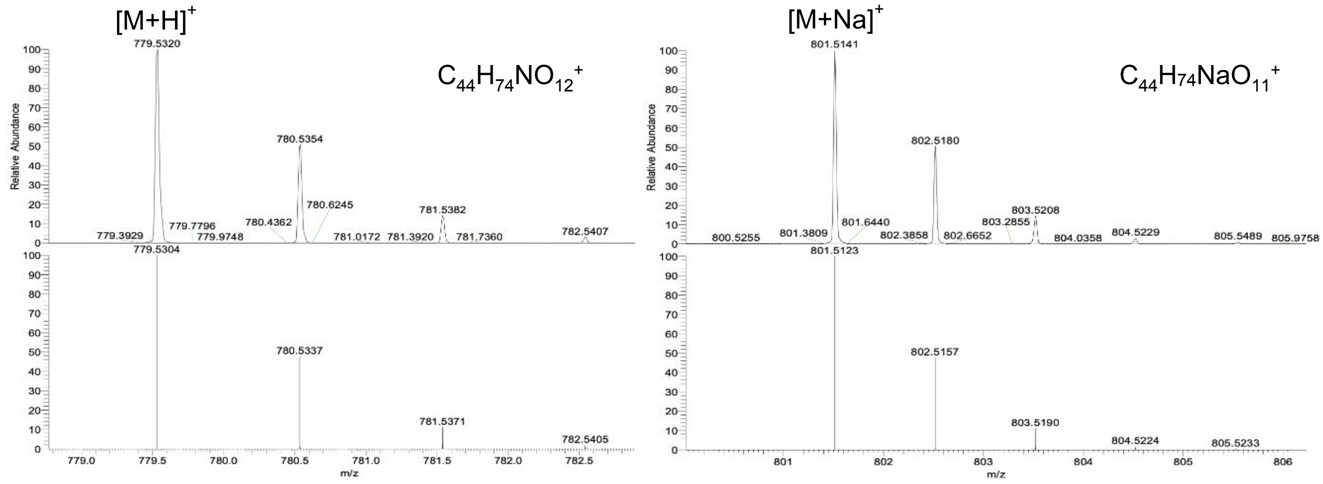

Figure S2. High resolution mass spectra of gladiostatin (F3) and gladiolin (F6). (A) Simulated (top) and measured (bottom) spectra for the $[M+H]^+$ and the $[M+Na]^+$ ions of gladiostatin (F3). **(B)** Simulated (top) and measured (bottom) spectra for the $[M+H]^+$ and the $[M+Na]^+$ ions of gladiolin (F6).

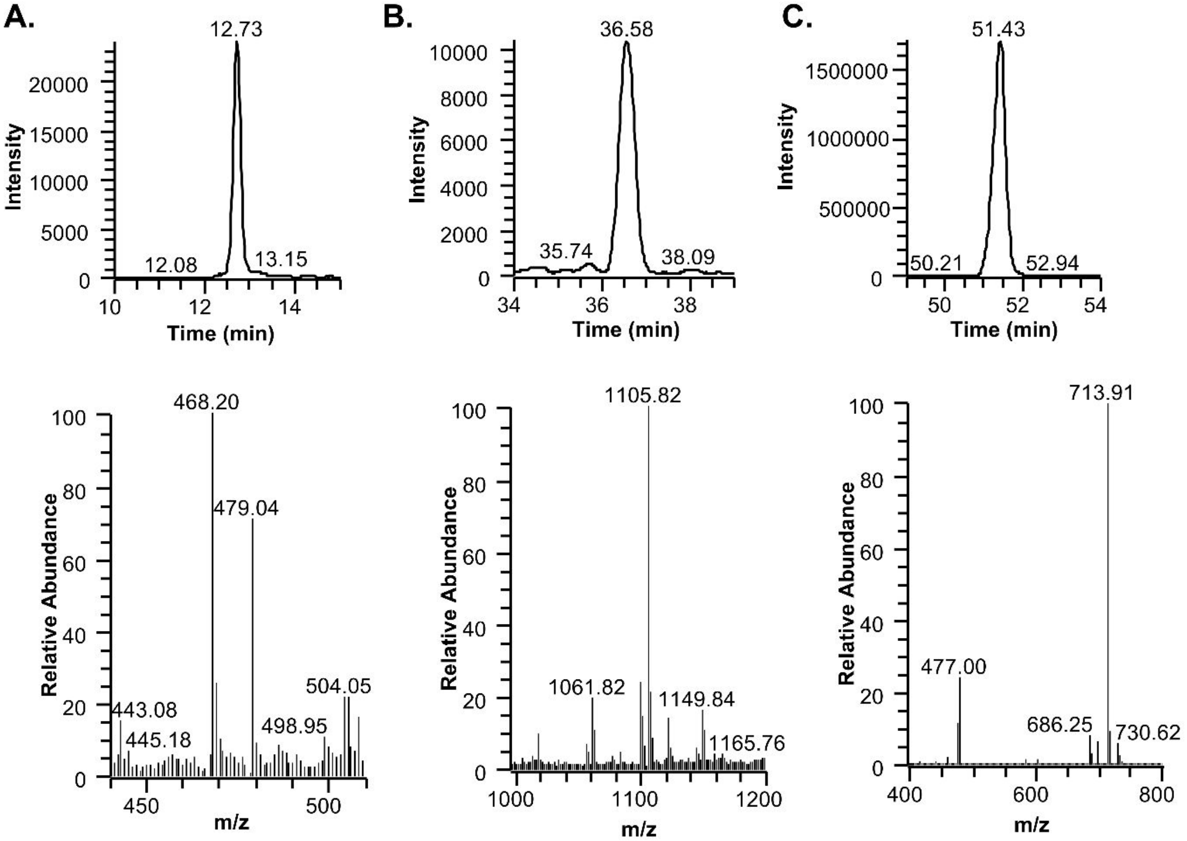

Figure S3. LC–MS detection of putative secondary-metabolite features in *B. gladioli* BBB-01 crude extract. Extracted-ion chromatograms (EICs) and corresponding full-scan mass spectra are shown for ions tentatively assigned (by precursor m/z and retention time) as sinapigladioside-like (m/z 468–469, ESI−; RT 12.73 min; m/z 468.20) **(A)**, plantaribactin/gladiobactin-like (m/z 1105–1106, ESI+; RT 36.58 min; m/z 1105.82 [M+H]+) **(B)**, and icosalide-like (m/z 713–714, ESI+; RT 51.43 min; m/z 713.91 [M+H]+) **(C)**. Full-scan data were acquired over m/z 250–2000.

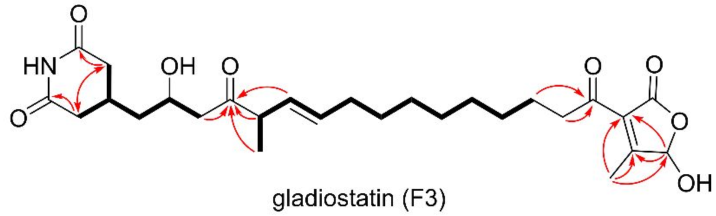

gladiostatin (F3)

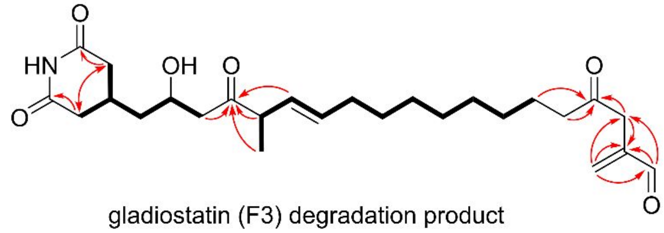

gladiostatin (F3) degradation product

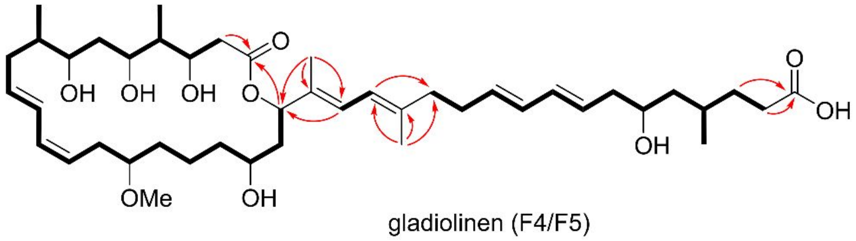

gladiolinen (F4/F5)

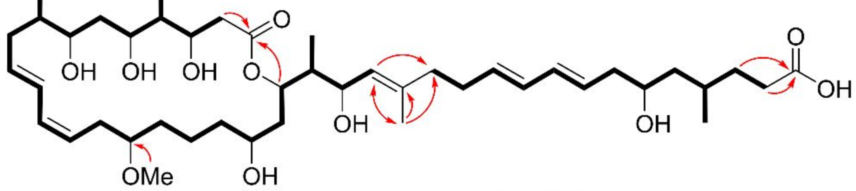

gladiolin (F6)

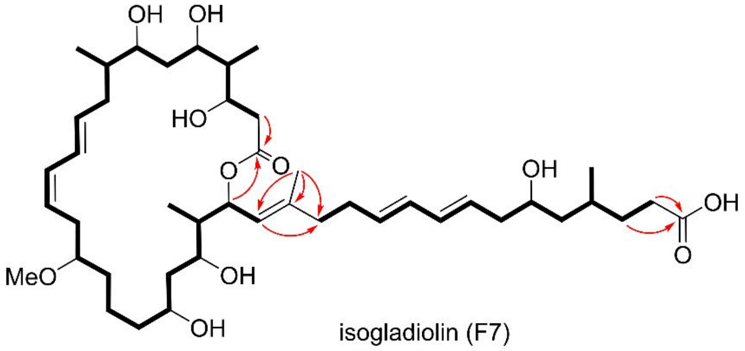

isogladiolin (F7)

COSY — HMBC

Figure S4. Key COSY and HMBC correlations of F3–F7. The planar structures of F3–F7 are constructed based on their COSY and HMBC correlations.

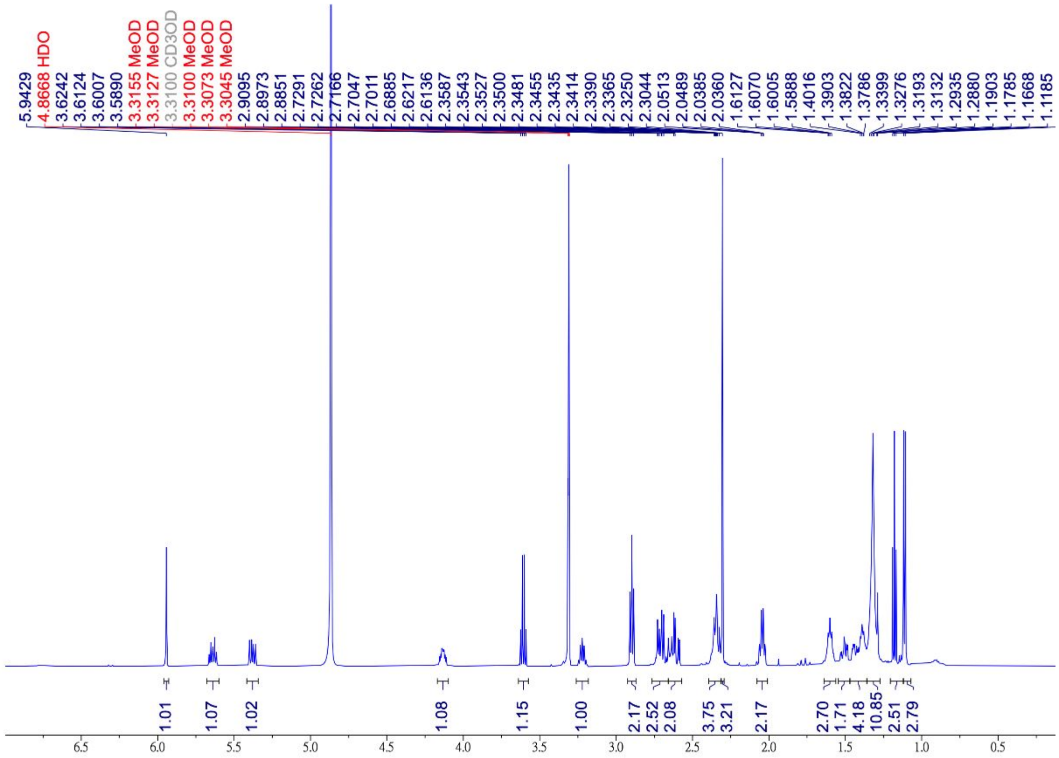

Figure S5. ^{1}H-NMR (600 MHz) spectrum of gladiostatin (F3) in CD_3OD.

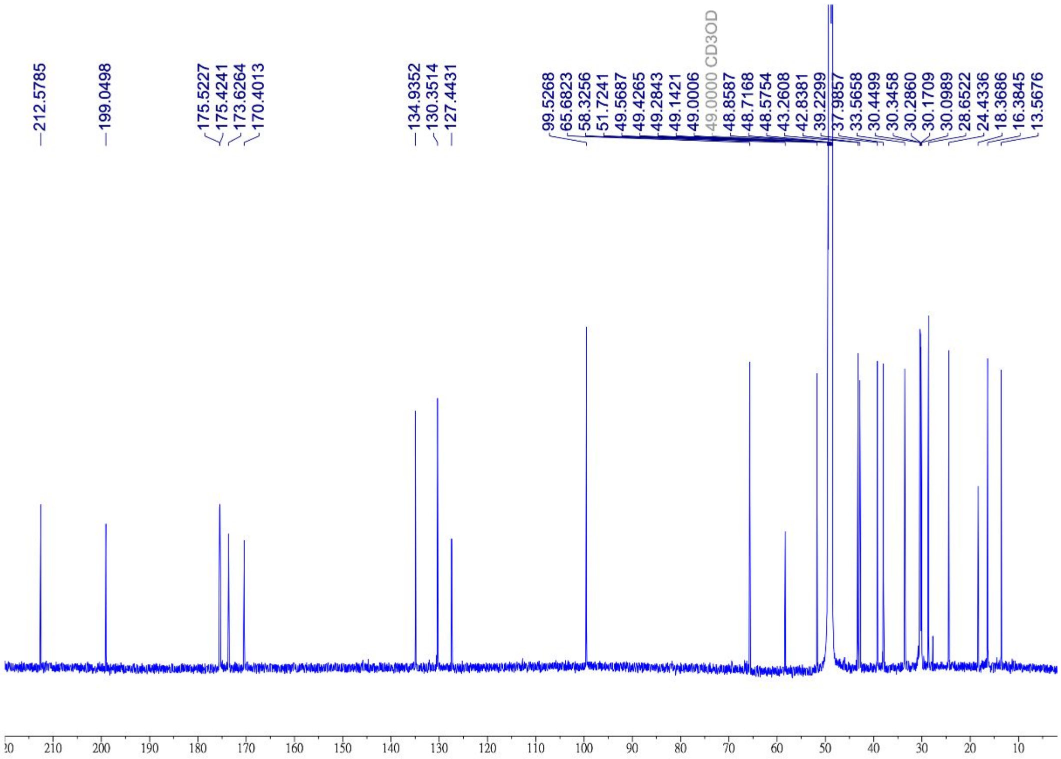

Figure S6. ^{13}C-NMR (150 MHz) spectrum of gladiostatin (F3) in CD_3OD.

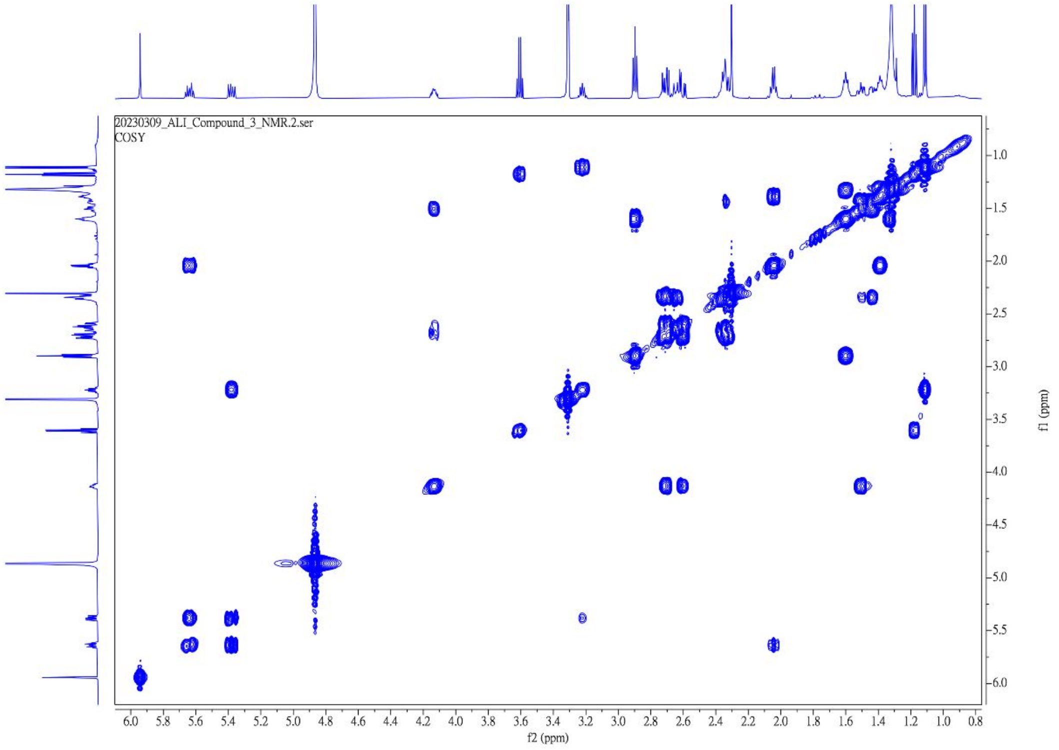

Figure S7. COSY spectrum of gladiostatin (F3) in CD_3OD.

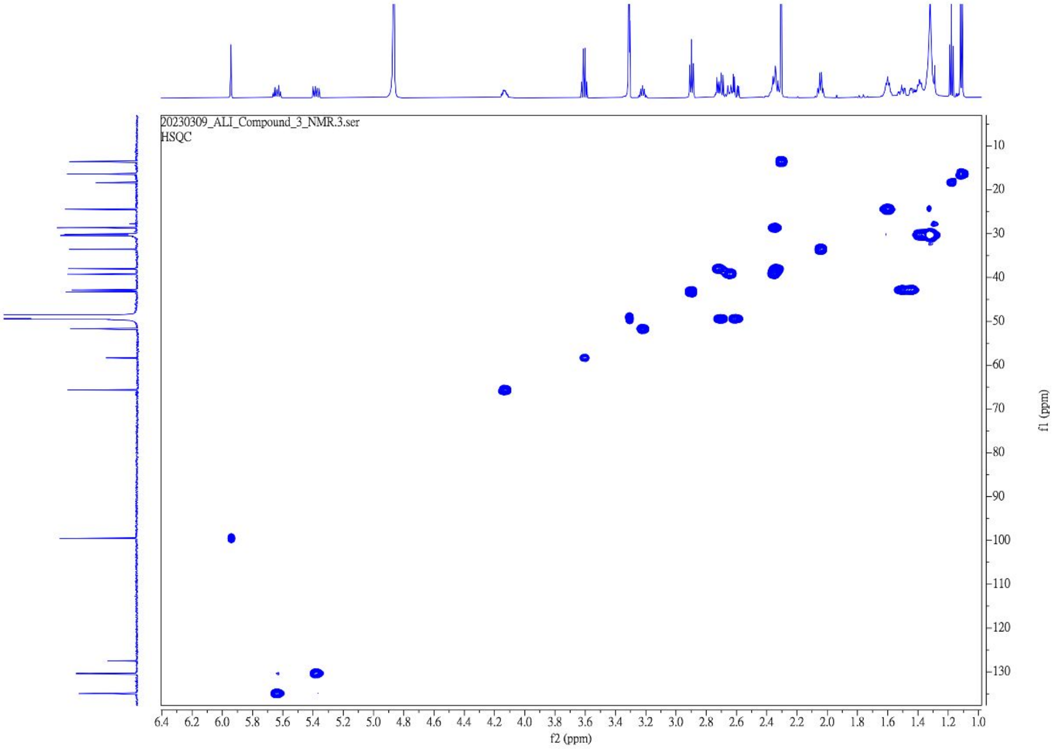

Figure S8. HSQC spectrum of gladiostatin (F3) in CD_3OD.

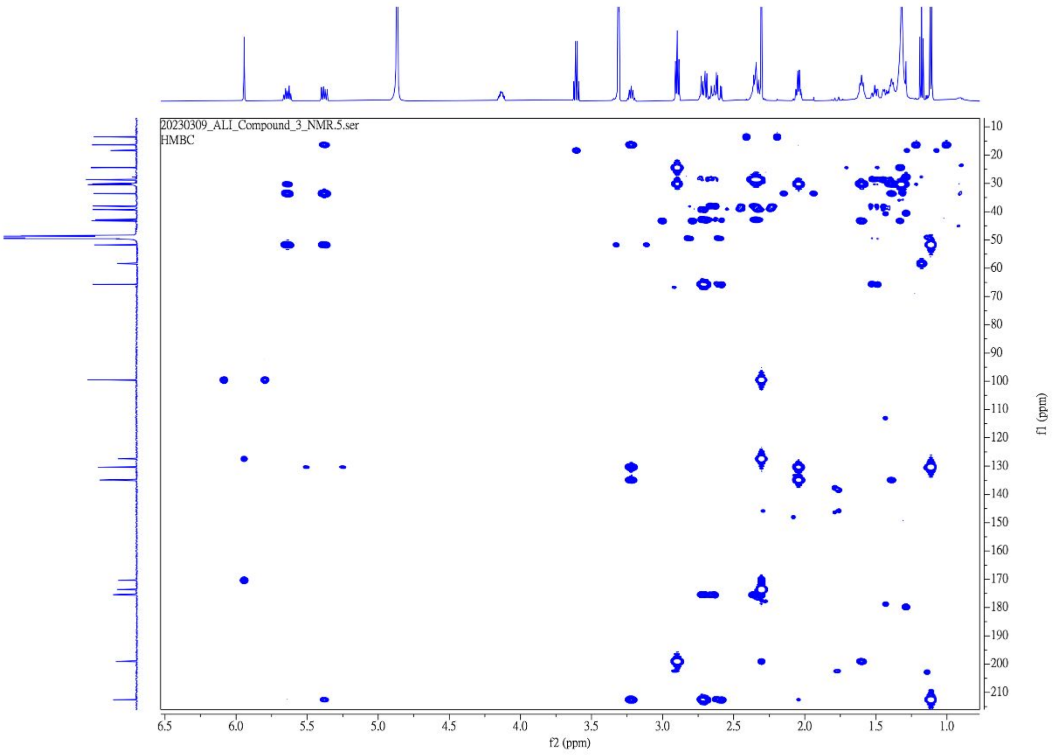

Figure S9. HMBC spectrum of gladiostatin (F3) in CD_3OD.

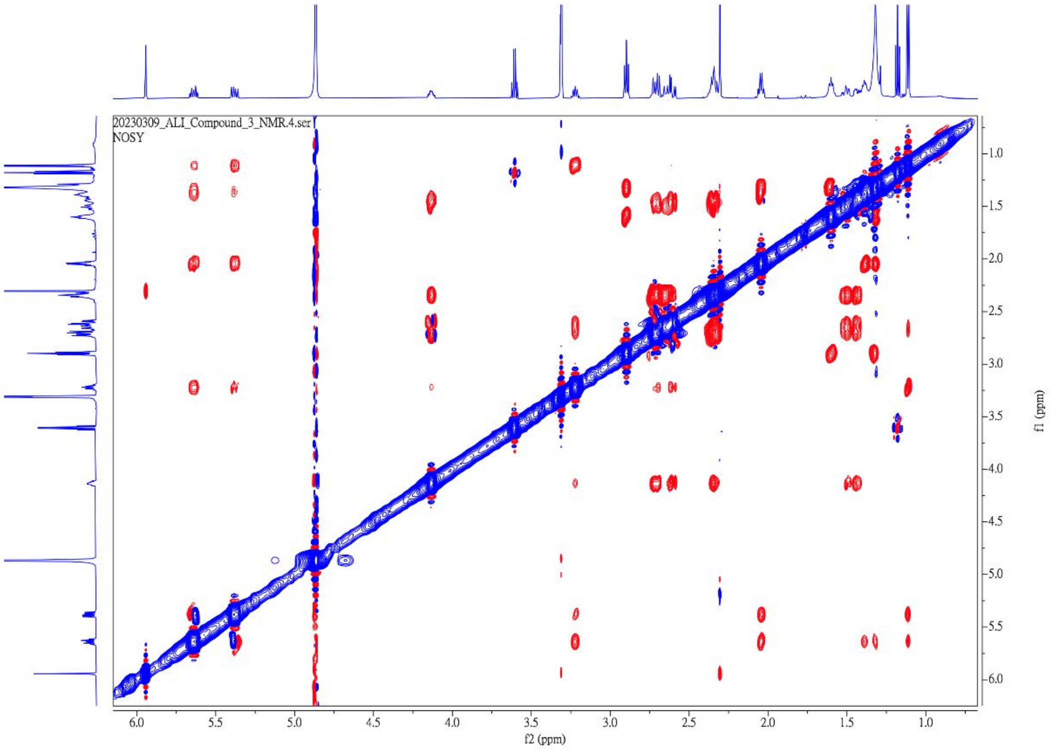

Figure S10. NOESY spectrum of gladiostatin (F3) in CD_3OD.

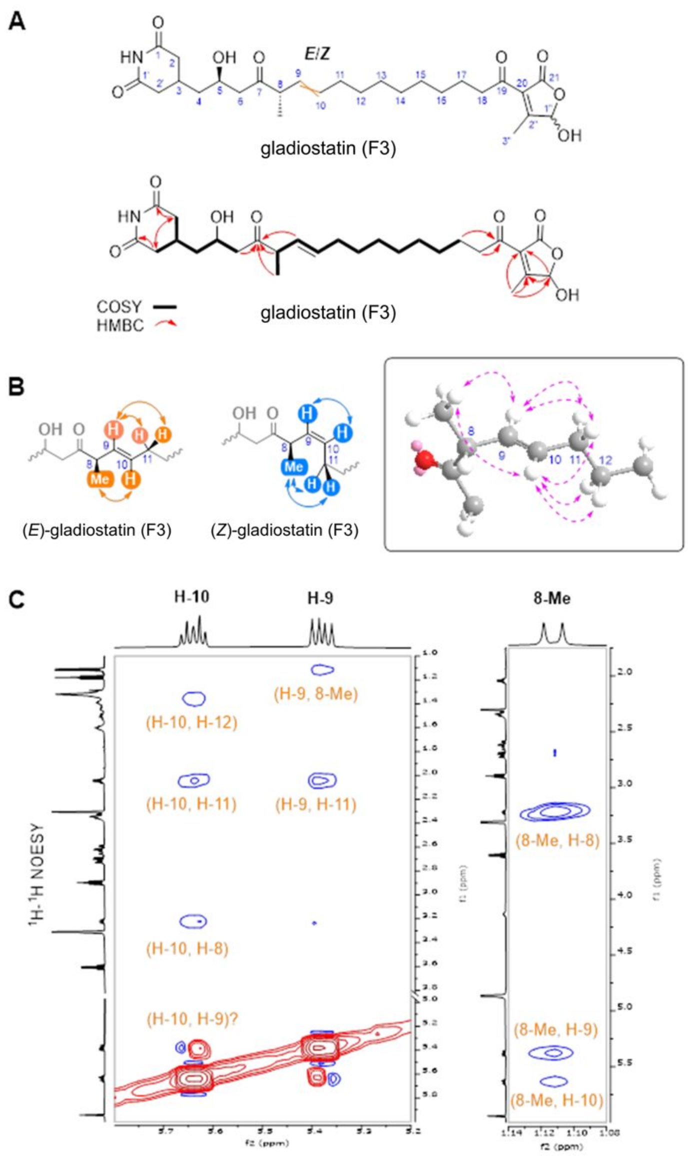

Figure S11. Geometric confirmation of gladiostatin (F3). (A) The chemical structure of gladiostatin and its planar structure is connected according to the 2D-NMR (COSY and key HMBC correlations) data. **(B)** Two configurations (E- and Z-forms) of double bonds of gladiostatin are illustrated (left), which are general in long chains and their interconversion, especially in fatty acids. The favor geometry (right) can de distinguished by the exact NOESY correlations (pink dashed double arrows). **(C)** Critical NOESY correlations between 8-Me, H-9, H-10, and their neighboring protons in the NOESY spectrum of gladiostatin (F3) are shown.

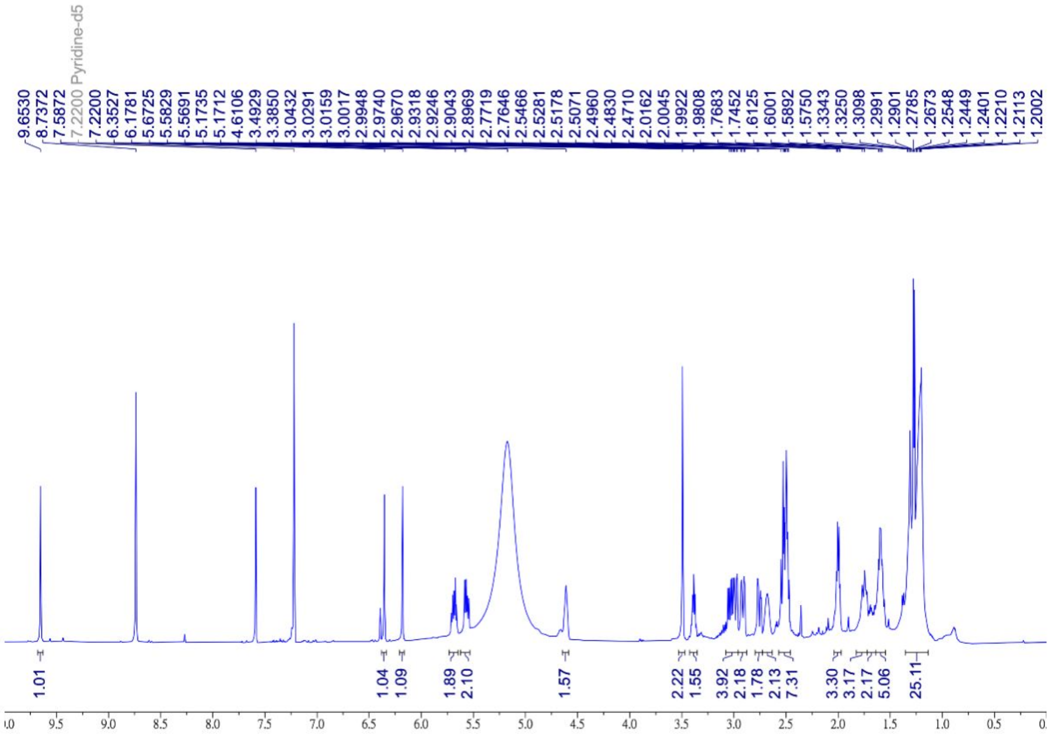

Figures S12. ^{1}H-NMR (600 MHz) spectrum of gladiostatin (F3) degradation product in pyridine-d_5.

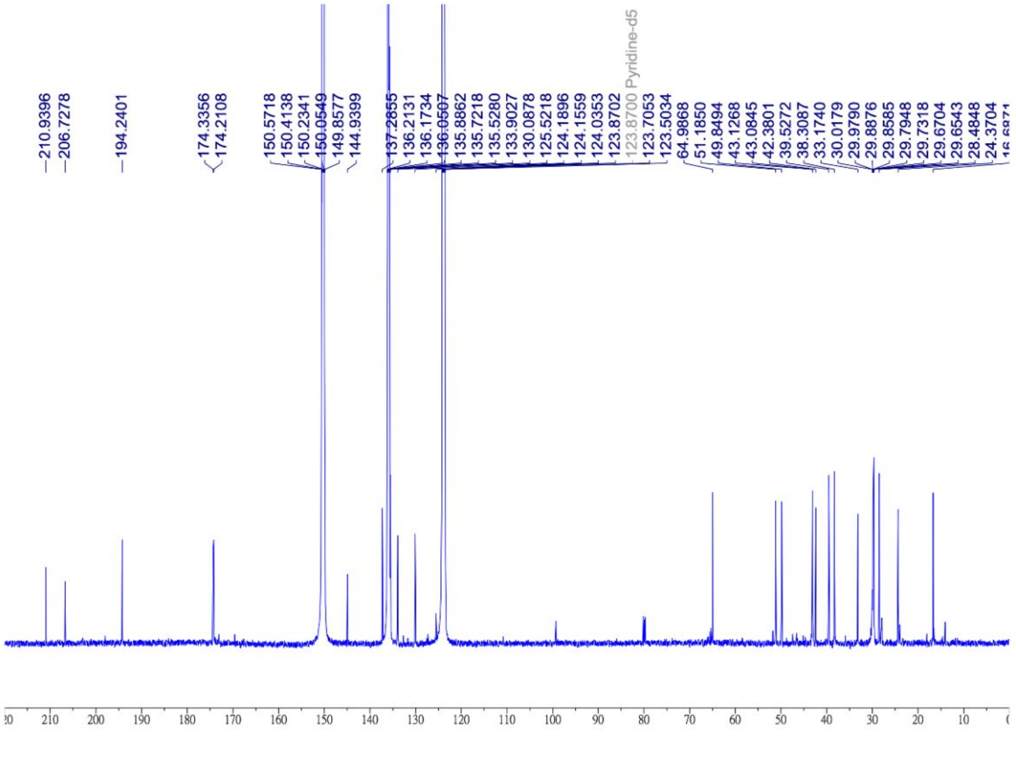

Figure S13. ^{13}C-NMR (150 MHz) spectrum of gladiostatin (F3) degradation product in pyridine-d_5.

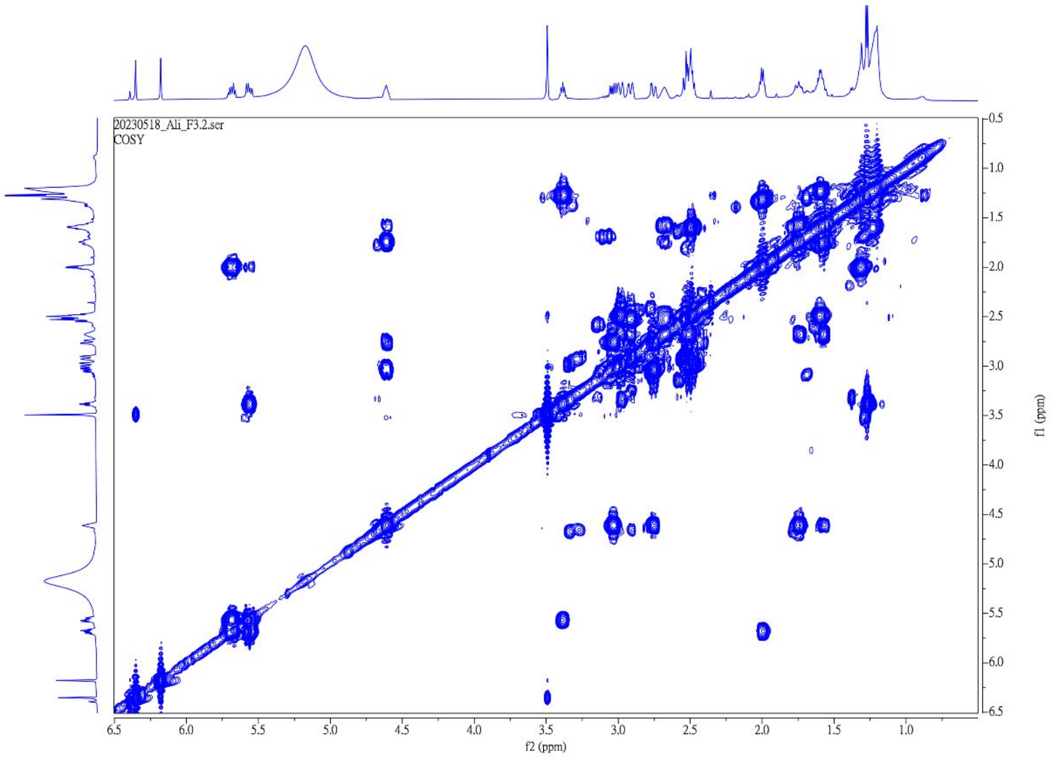

Figure S14. COSY spectrum of gladiostatin (F3) degradation product in pyridine-d_5.

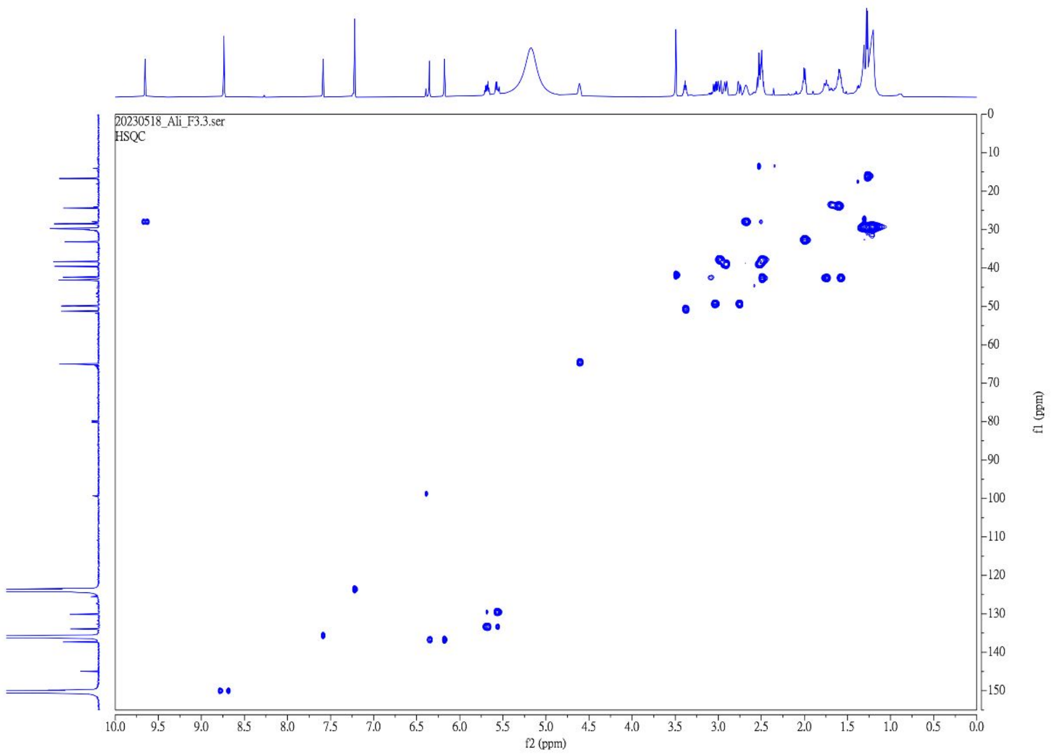

Figure S15. HSQC spectrum of gladiostatin (F3) degradation product in pyridine-d_5.

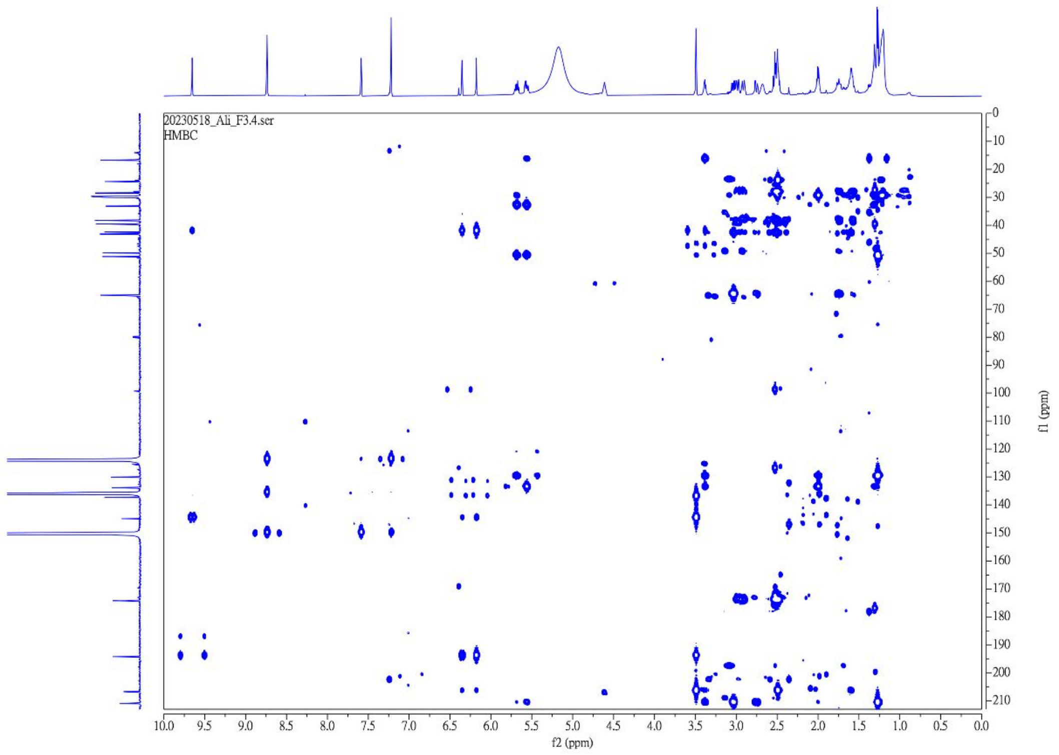

Figure S16. HMBC spectrum of gladiostatin (F3) degradation product in pyridine-d_5.

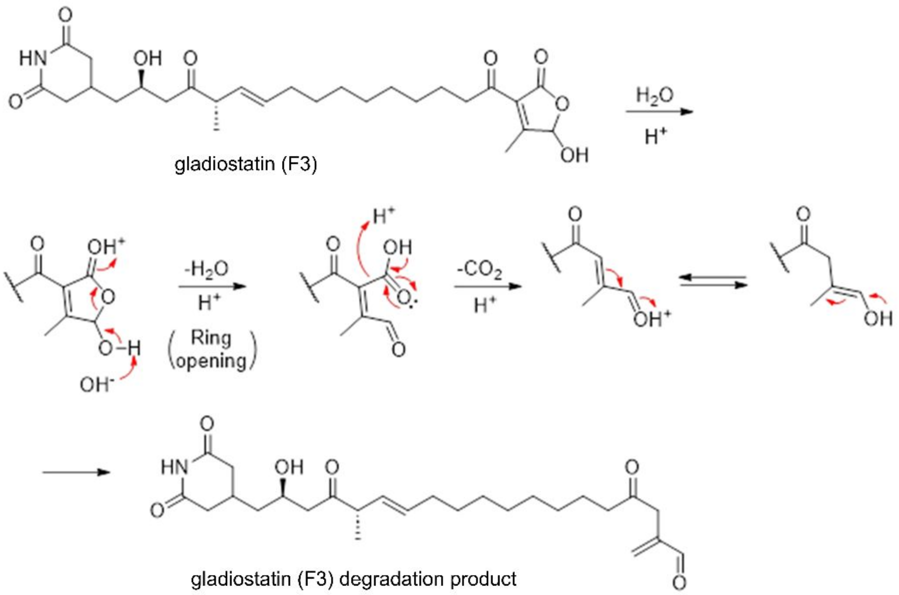

Figure S17. Proposed mechanism for the conversion of gladiostatin (F3) to a degradation product.

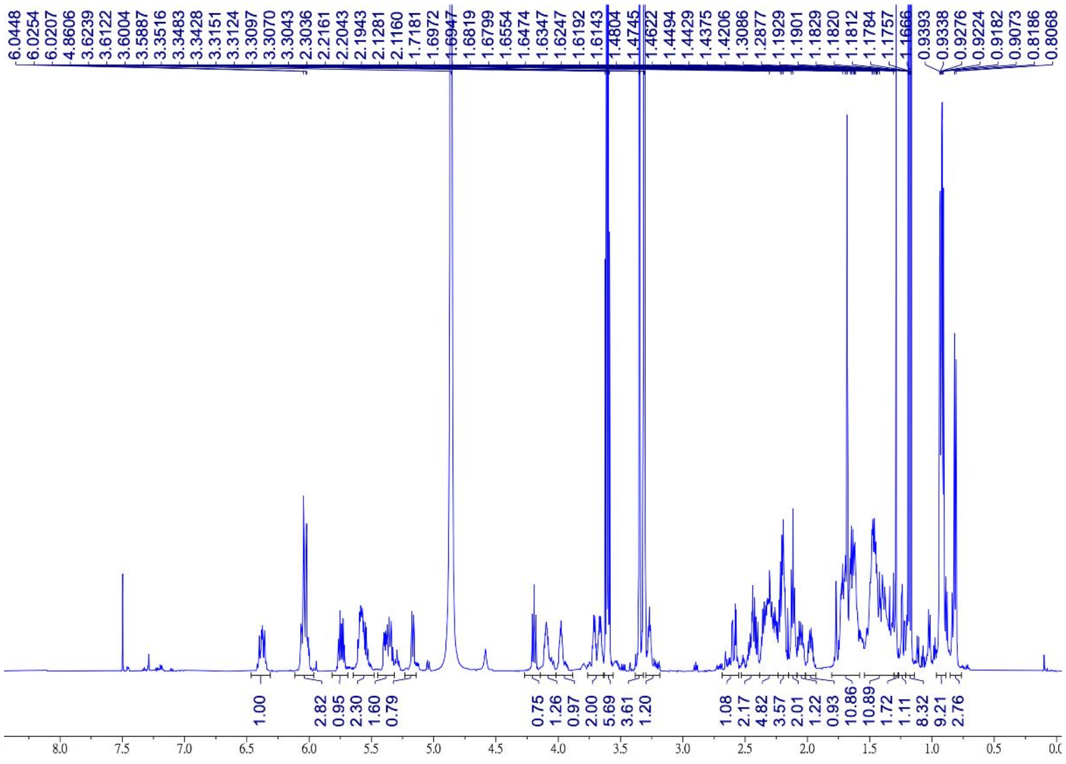

Figure S18. ^{1}H-NMR (600 MHz) spectrum of gladiolin (F6) in CD_3OD.

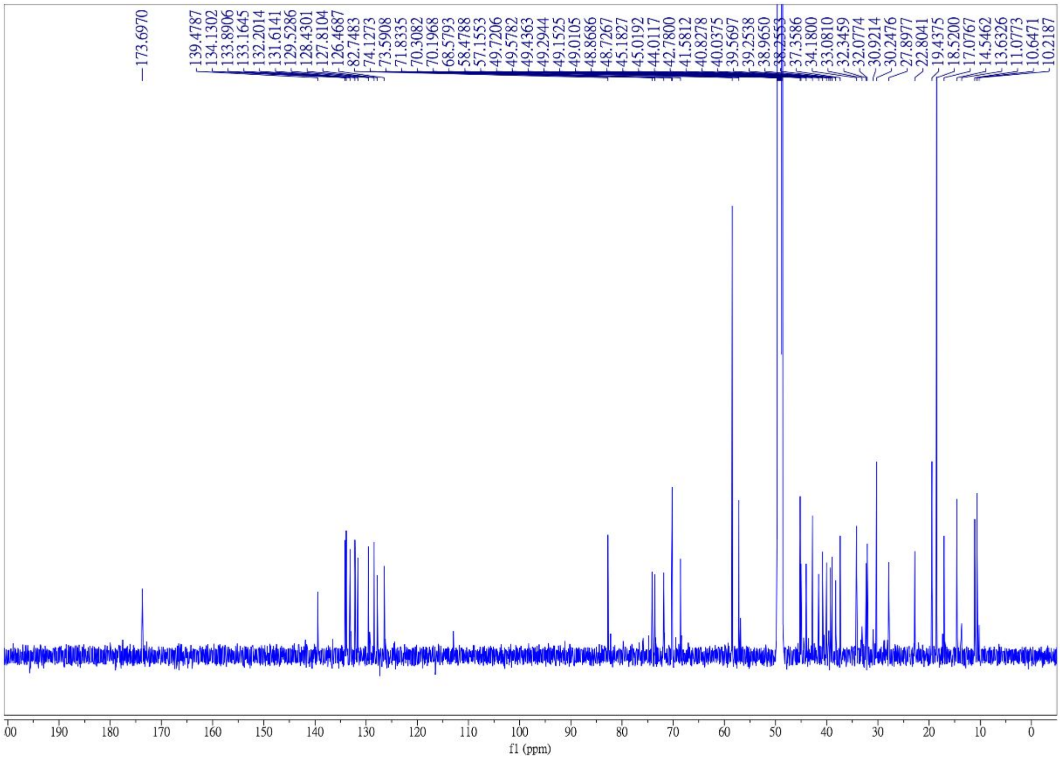

Figure S19. ^{13}C-NMR (150 MHz) spectrum of gladiolin (F6) in CD_3OD.

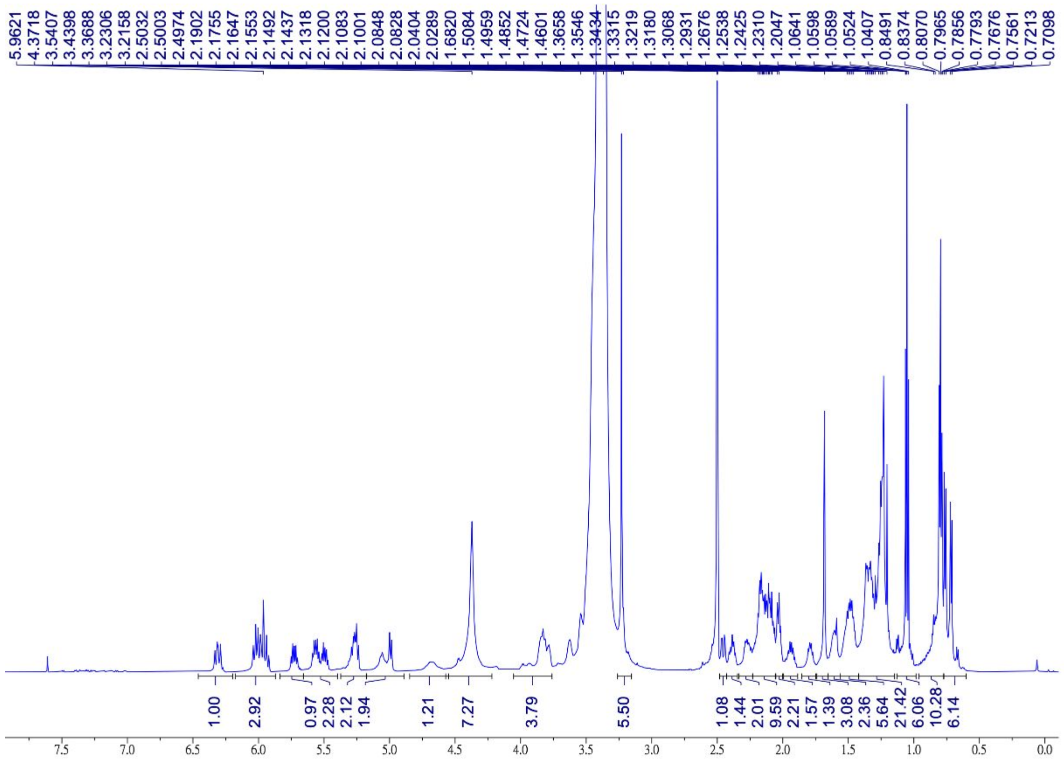

Figure S20. ^{1}H-NMR (600 MHz) spectrum of isogladiolin (F6) in DMSO-d_6.

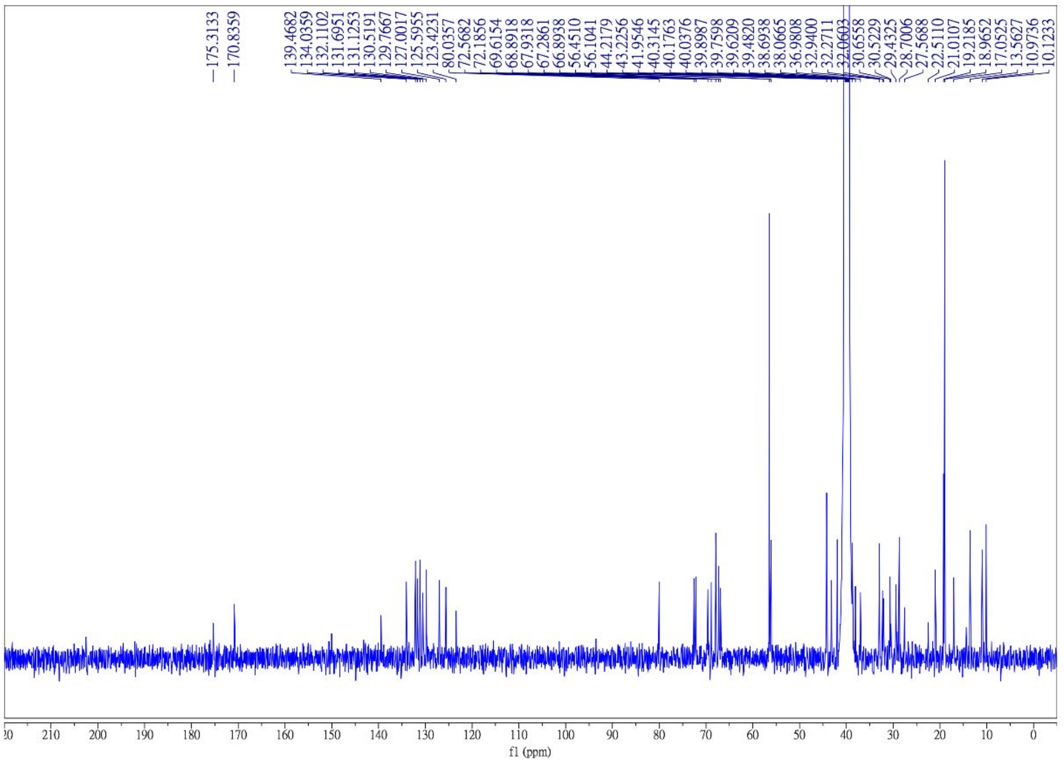

Figure S21. ^{13}C-NMR (150 MHz) spectrum of isogladiolin (F7) in DMSO-d_6.

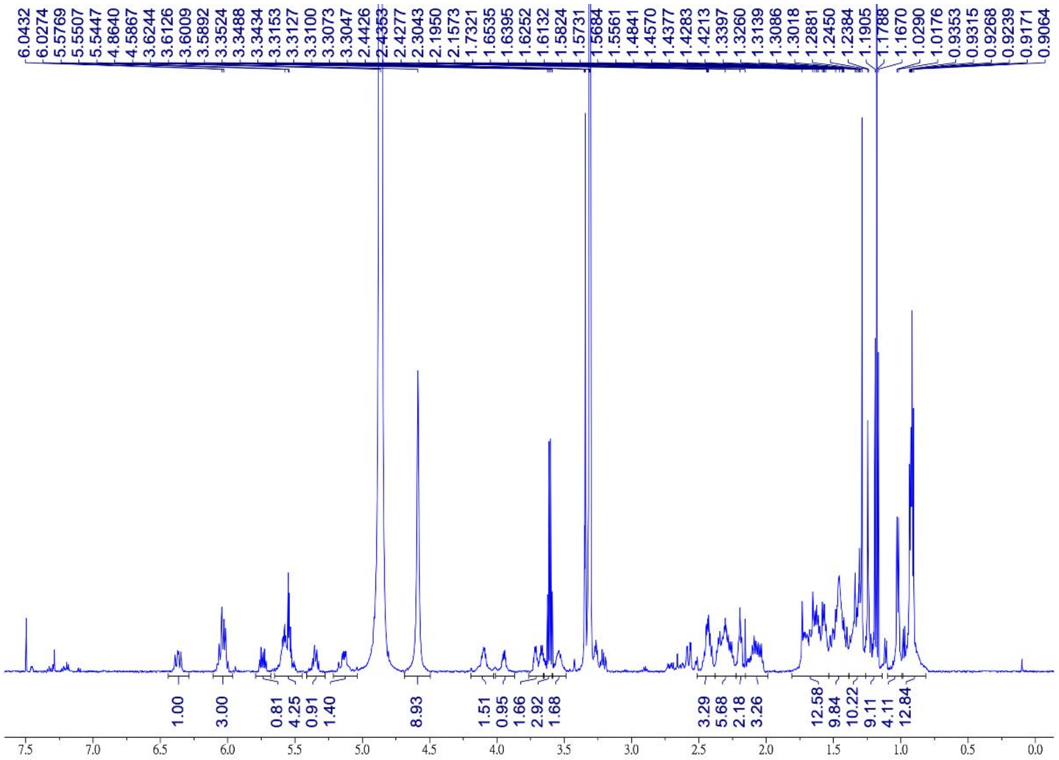

Figure S22. ^{1}H-NMR (600 MHz) spectrum of F4 in CD_3OD.

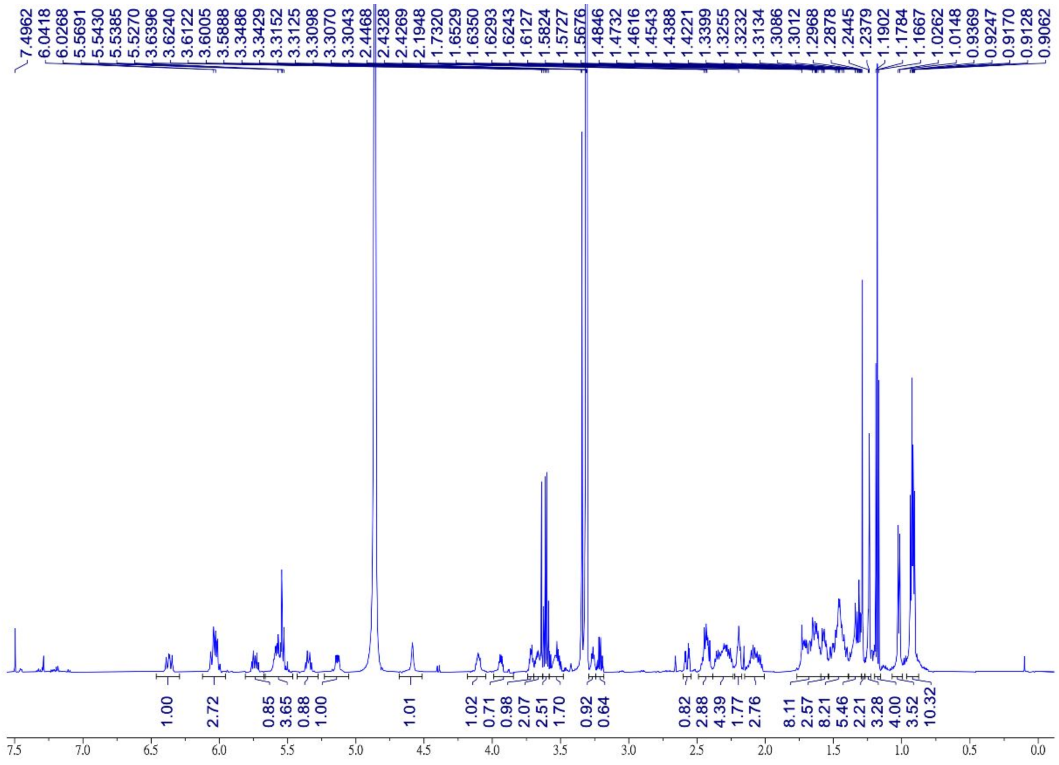

Figure S23. ^{1}H-NMR (600 MHz) spectrum of F5 in CD_3OD.

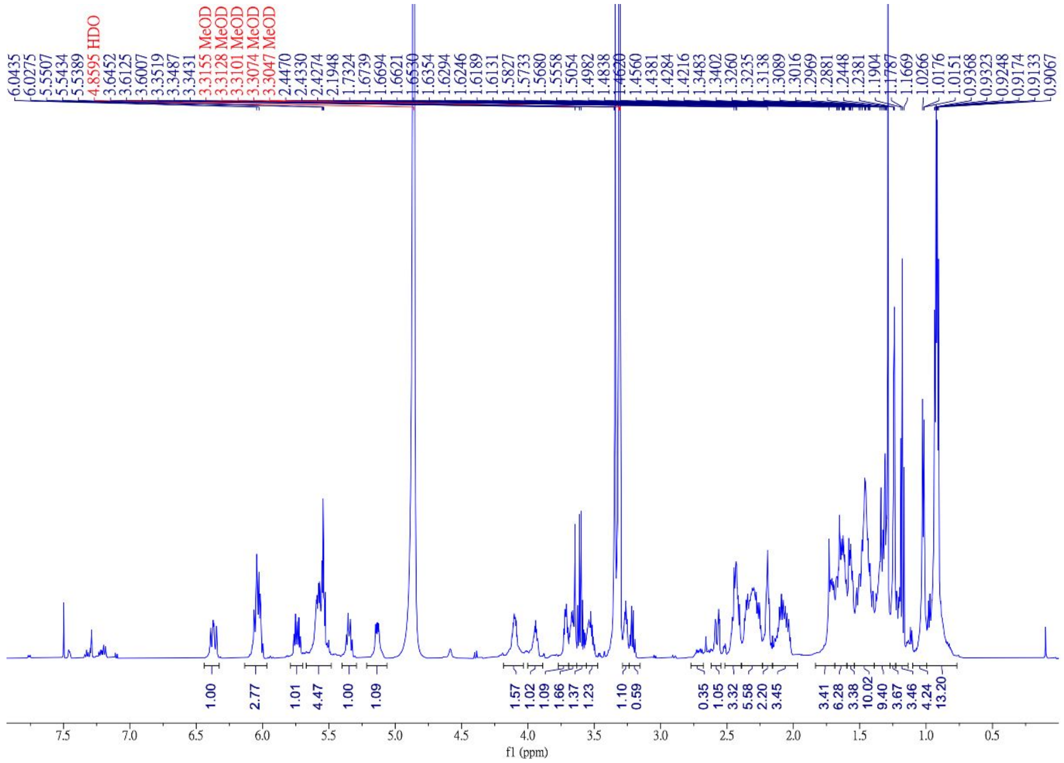

Figure S24. ^{1}H-NMR (600 MHz) spectrum of gladiolinen (F4/F5) in CD_3OD.

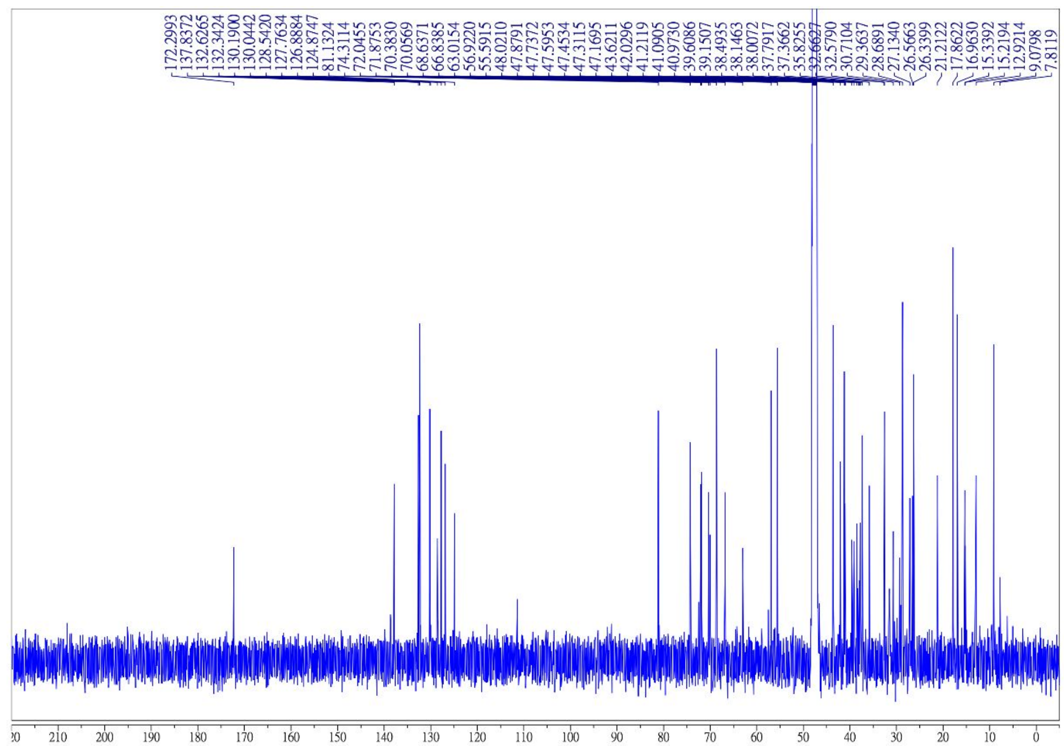

Figure S25. ^{13}C-NMR (150 MHz) spectrum of gladiolinen (F4/F5) in CD_3OD.

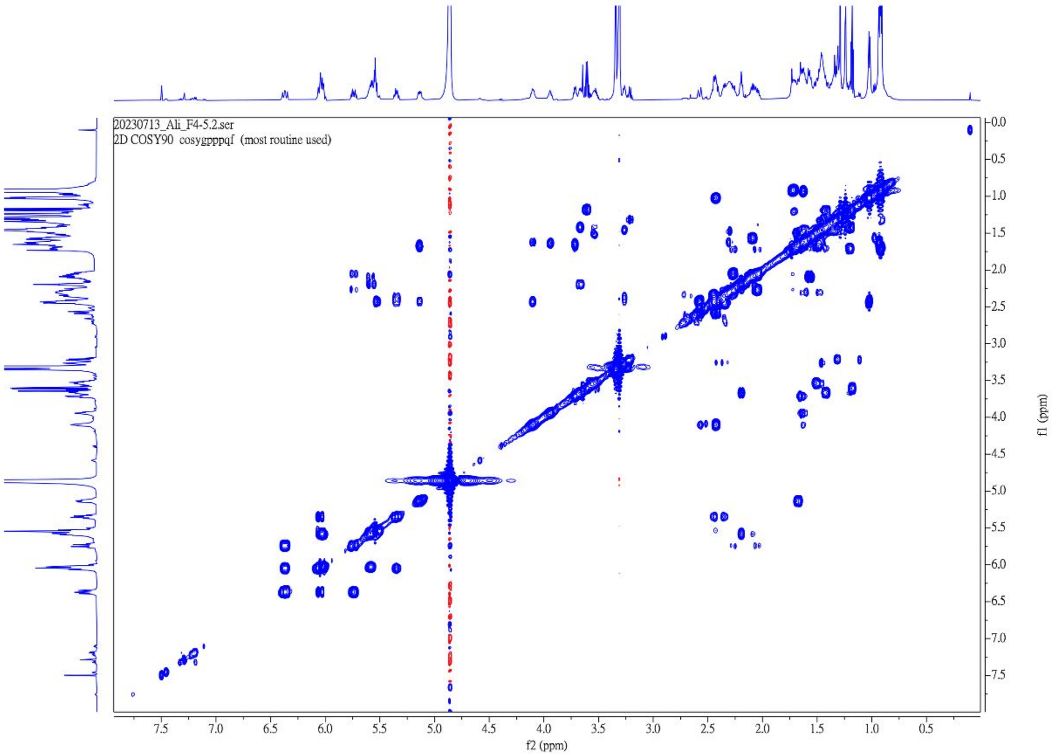

Figure S26. COSY spectrum of gladiolinen (F4/F5) in CD_3OD.

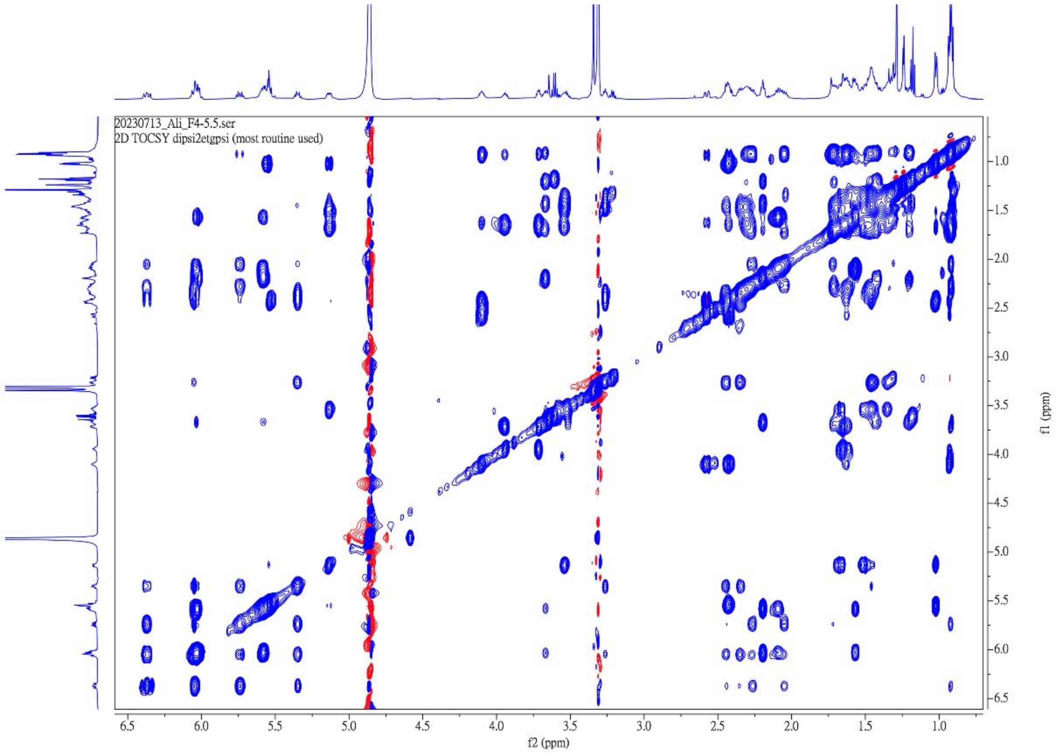

Figure S27. TOCSY spectrum of gladiolinen (F4/F5) in CD_3OD.

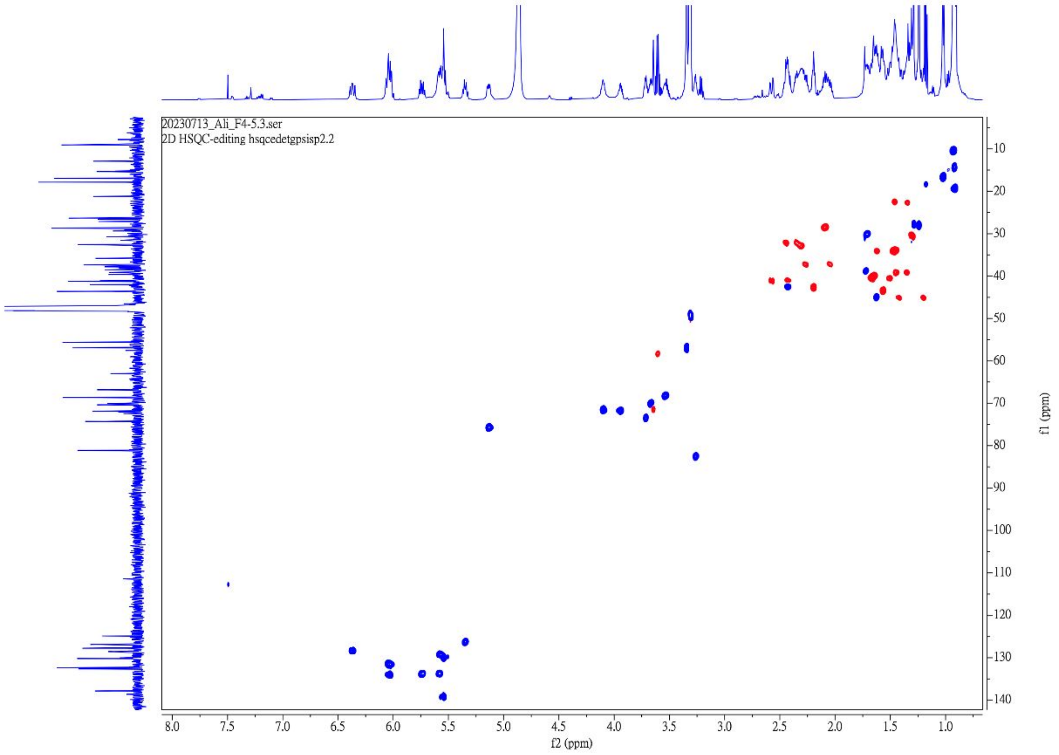

Figure S28. HSQC spectrum of gladiolinen (F4/F5) in CD_3OD.

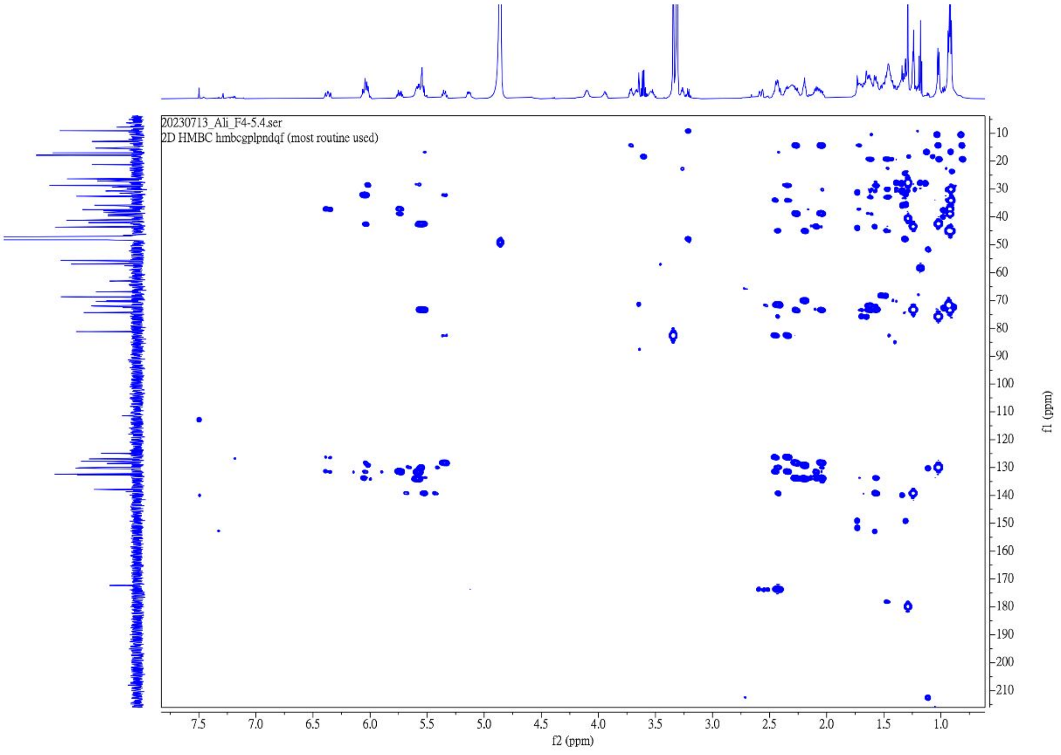

Figure S29. HMBC spectrum of gladiolinen (F4/F5) in CD_3OD.

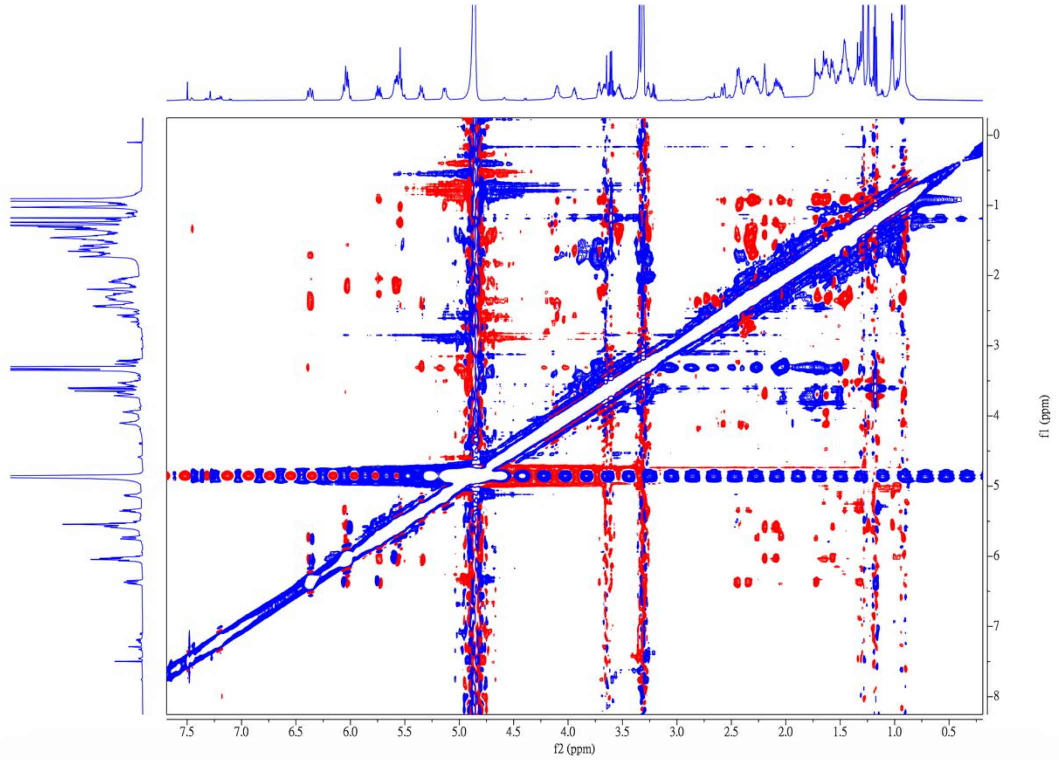

Figure S30. NOESY spectrum of gladiolinen (F4/F5) in CD_3OD.

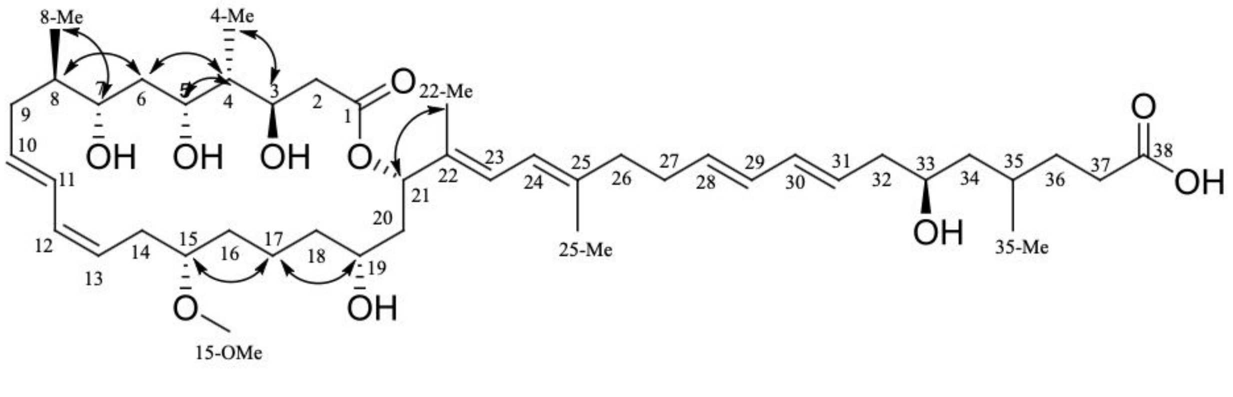

Figure S31. Key NOE correlations observed in the NOESY spectrum, mapped onto the proposed structure of gladiolinen.

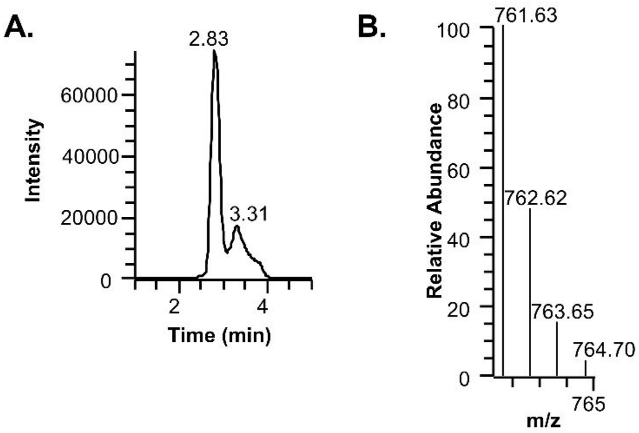

Figure S32. Chiral LC–MS analysis of the F4/F5 fraction. (A) EIC for the [M+H]+ ion at m/z 761–762 acquired on a chiral stationary phase, showing a single elution region with two overlapping local maxima at ~2.83 and ~3.31 min (no baseline separation). **(B)** Mass spectrum averaged across the elution region shown in panel A, confirming a single molecular ion cluster. The absence of baseline separation on the chiral column is consistent with closely related structural or stereochemical features that are not resolved under these conditions.

CCTGTTAAAAATACAGGCTAAATCCTCTCATTAATCGGTTGACTGGCTTCCCGATTTTATTGAACAATTTACCGCGCCGTGGGTA

lux box -35 -10

TTACATAATTCCGACCCGAAGCT**GATC**CGACAGCGTTCGACGGGTGC**TCGCAA**CACGGCAATTACACAAG**CAAAAT**CATCGAACG

Bga_0390F

GGCCGACAAGCTATGAACCAGATACAAACCATGAGGGTGTTCGTCTGCGTCGCCGAACTGCAGAGTTTCCGGCAGGCCGCGCGCA

AGCTCGGCGTGTCGAACGCACTCGTCACGCGGTCGATTGCGATGCTGGAAACGCATCT [GAACACTCGCCTGATCCATCGCACCA

CGCGCAATCTGTCGCTGACCGAGGCGGGTACCCGCTATCTCGACGGTTGCCGCGCCCTGCTCGAGGAATTCGATCATCTCGAGGC

CTCGGTCGCGCATACCATTCGCGAGCCGGTCGGCACCTTGCGGATCGTGGTGTCGGGCTCGCTGTCGCCGCAGCGACTCACGCCG

CTGGTCGACGGCTTCCGCCGCCAGTACCCCAAGGTGCGCGTGCAACTGACCGTCGCCGACGGCCCGATCGACGGGCTCGACACCG

CCTACGACGCGGCCATCATCGCCGGGCGCAAGATCGAGGACGGCGCGGCGCTGGTCAGCCACGCGCTCGCCCCCGATCCGTTCGT

CGCGGTCGCATCGCCGGCCTACATCGAGCAGCGCGGCGAGCCGCATCGCCCGGACGAGCTCGCGCGCCACGCCGCGGTCACGCTG

CCGCTCGACTCGCATGGTTTCGCCTGGCGCTTCGTCGACGCGGGCCGCTTCGCGCACCTGGTCACGCTGC] AGCCGGCCTACACC

ATCAACGACGCCTGGCTGGTGCGCGCCGCCGTGATGGCCGGCTCCGGCATCGCGATCCTGCCCGAGAGCTTCGTGGCCGACGCGA

TCTCGCGCGGCGAGCTGGTTCGCCTGCTGGCCGACTACCGGATCGACGATACCGACGCGCAGCTCGCGGTGGTCTATCCGAACCG

Bga_0390R

CCAGTTCGTGCCGGCGCGCACGCGCAGCTTCGTCGAGCACGCGCTCTACCATTTCGGCGCGCGCTCGAACGGCCGCTACGGCTAC

GTGCACGACGCGATCGGCGAGCGCAGCGAGGTGGCCACCGGCCTGCAGTGATCGCGTGCGAGGACGGCCGGCGCC...........

Figure S33. The nucleotide sequence of the promoter region and open-reading frame of *lttr_0390*. The predicted lux box is underscored with a yellow background with the conserved GATC in bold. The ORF sequence of *lttr_0390* is colored red, within which the 521 nucleotides enclosed by the bracket are replaced by the *tet*^{R} expression cassette in the Δ0390 knockout strain. The matched region of primers Bga_0390F and Bga_0390R, used in PCR to confirm the insertion of the *tet*^{R} expression cassette in *lttr_0390*, are indicated with blue arrows.

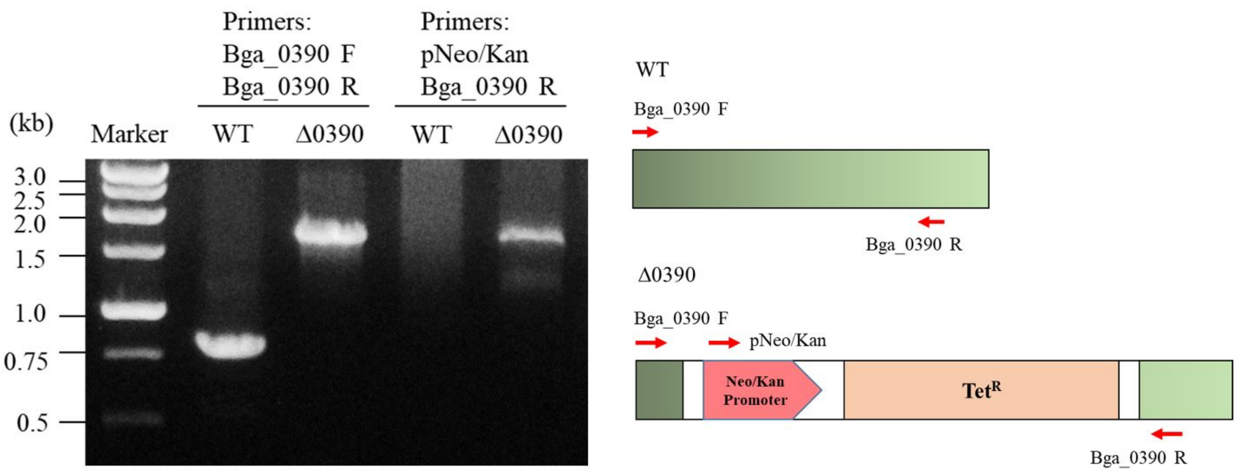

Figure S34. Confirmation of *lttr_0390* disruption by PCR. PCR was performed using primers Bga_0390 F and Bga_0390 R or primers pNeo/Kan and Bga_0390 R. As anticipated, the primer pair Bga_0390F and Bga_0390R amplified ~0.8 kb and ~1.7 kb DNA fragments from the WT and Δ0390 chromosome templates, respectively. The DNA regions matched by the primers are illustrated in the right panel scheme.

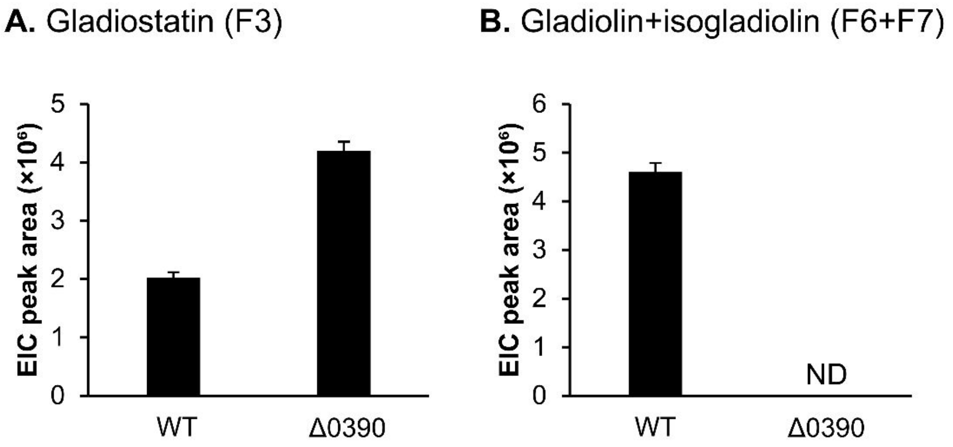

C.

Compound	WT EIC area (mean ± SD)	Δ0390 EIC area (mean ± SD)	Fold-change (Δ0390/WT)	WT (µg eq/mg crude)	Δ0390 (µg eq/mg crude)
**F3 (gladiostatin)**	$(2.02 \pm 0.10) \times 10^6$	$(4.20 \pm 0.16) \times 10^6$	2.08	142.3 ± 7.0	295.9 ± 11.3
**F6+F7 (gladiolin + isogladiolin)**	$(4.60 \pm 0.18) \times 10^6$	ND	—	342.1 ± 13.7	ND

Footnote: Crude extracts were analyzed in ESI+ full-scan mode (200 µg crude injected per run; n = 3). EIC peak areas were integrated using fixed m/z and retention-time windows. Semi-quantitative equivalents (µg eq/mg crude) were estimated by single-point external calibration using purified standards analyzed under the same method: F3 (25 µg; mean area $(1.78 \pm 0.07) \times 10^6$, n = 3) and F6 (20 µg; mean area $(1.35 \pm 0.08) \times 10^6$, n = 3). Because F6 can partially interconvert to its isomer (F7) during handling, F6/F7 values are reported as approximate F6-equivalents. ND, not detected in the specified EIC/RT window.

Figure S35. LC–MS EIC–based comparison of F3 and F6+F7 in WT versus Δ0390 crude extracts. **(A)** F3 EIC peak area; Δ0390 differed from WT (Welch's two-tailed t-test, $p = 1.35 \times 10^{-4}$). **(B)** Ion-summed EIC peak area for F6+F7; signal was detected in WT but ND in Δ0390. For WT, the two resolved peaks (F6 and F7) were summed. **(C)** Summary of EIC areas, fold-change, and semi-quantitative equivalents. Bars show mean ± SD (n = 3 injections).

Table S1. Microorganisms used in this study.

Microorganism	Source	Reference/Obtained
B. gladioli BBB-01	Healthy rice shoots	[1]
B. cepacia Xitou	Urban soil	[1]
S. aureus	Human excretions	This study
P. aeruginosa	Human excretions	This study
K. pneumoniae	Human excretions	This study
R. solanacearum	Wilted tomato stems	This study
M. grisea 14008	Infected rice shoots	This study
M. oryzae MORY-9	Infected rice shoots	This study
C. violaceum CV026	-	[2]
B. cereus	-	BCRC[#]
C. albicans	-	BCRC[#]
C. glabrata	-	BCRC[#]
C. parapsilosis	-	BCRC[#]
S. cerevisiae INVSc1	-	Invitrogen (Carlsbad, CA)
E. coli BL21	-	New England Biolabs (Ipswich, MA)

[#]BCRC: Bioresource Collection and Research Center, Hsinchu, Taiwan.

Table S2. The nucleotide sequence of primers used in this study.

Primer Name	Sequence (5'→3')	Purpose
p70-tet vector F	ACCACTCCAAGAGCGGGACTCTGGGGTTCGAAAT	Construction of pBBR1-Rha-ETh1h2e_yi23-tet
p70-tet vector R	GTTAGATTTCATGCGAAACGATCCTCATCCTGTCTC	
p70-tet gene F	GGATCGTTTCGCATGAAATCTAACAATGCGCTCATCGTCAT	
p70-tet gene R	CCAGAGTCCCGCTCTTGGAGTGGTGAATCCGTTAG	
Bga_0390 p70 gene F	GGAGGATATCCTATGAACCAGATACAAACCATGAGGGTGT	Construction of pBBR-0390
Bga_0390 p70 gene R	GCACACGGTCACATTGCGGCTATTGGCCGCAT	
Bga_0390 p70 vector F	AATAGCCGCAATGTGACCGTGTGCTTCTCAAATG	
Bga_0390 p70 vector R	GTATCTGGTTCATAGGATATCCTCCTTCTTAAGAATTGTTCA	
Bga_0390 F	ATGAACCAGATACAAACCATGAGGGTGTTC	Verification of the insertion of *tet*^{R} in the chromosomal *LTTR_0390*
Bga_0390 R	TCGGTATCGTCGATCCGGTAGT	
pNeo/Kan	CTTACATGGCGATAGCTAGACTG	
Bga_0390 Left arm R	CAGTCTAGCTATCGCCATGTAAGAGATGCGTTTCCAGCATCGCAAT	Construction of the overlap extension PCR fragment *LTTR_0390::tet*^{R}
Bga_0390 Right arm F	CTAACGGATTCACCACTCCAAGAAGCCGGCCTACACCATCAA	
Bga_0390 Tet overlap F	ATTGCGATGCTGGAAACGCATCTCTTACATGGCGATAGCTAGACTG	
Bga_0390 Tet overlap R	TTGATGGTGTAGGCCGGCTTCTTGGAGTGGTGAATCCGTTAG	

Table S3. The putative BGCs for secondary metabolite production in the genome of *B. gladioli* BBB-01.

Region	Type	Nucleotide range	Most similar known cluster	Similarity confidence
**Chromosome 1 (NCBI Reference Sequence: NZ_CP068049.1)**				
1.1	T1PKS	765290~812948	capsular polysaccharide	low
1.2	RiPP-like, NRPS	1602581~1666607		
1.3	isocyanide	1986167~2028002	sinapigladioside	high
1.4	polyyne	2063733~2090065	caryoynencin	high
1.5	NRPS, T1PKS	2222814~2362613	syringomycin	low
1.6	terpene	2637812~2658651		
**Chromosome 2 (NCBI Reference Sequence: NZ_CP068050.1)**				
2.1	transAT-PKS	3849395~21645	gladiostatin	high
2.2	terpene	500517~521401		
2.3	NRPS	1010286~1094613	plantaribactin	high
2.4	NRPS	1106276~1163219	haereogladiodin	high
2.5	cyanide	1194911~1207937		
2.6	RiPP-like	1246510~1257526		
2.7	phosphonate	1517217~1551691		
2.8	hserlactone	1802574~1823188		

2.9	NRPS	1867980~1891310	burriogladiodin	high
2.10	trans-AT-PKS	2135008~2275195	lagriene	high
2.11	terpene, RiPP, β-lactone	2947232~2989129		
2.12	NRPS	3113128~3168178	icosalide A/B	high
2.13	ectoine	3172462~3182848		
2.14	terpene	3213476~3234528		
2.15	terpene	3349687~3373139		
2.16	redox-cofactor	3418897~3441123		
g2.17	terpene	3514368~3535438		

Footnote: BGC regions were predicted by antiSMASH (v8.0.4; Jan 10, 2026) and summaried by manual analysis. Chromosome 2 is circular and was deposited as a linear sequence with an arbitrary breakpoint that falls within the gladiostatin BGC; therefore, Region 2.1 is reported as 3,849,395~21,645, meaning the cluster extends from 3,849,395 to the end of the deposited sequence and continues from the beginning to 21,645. The assignment of Region 2.4 as the haereogladiodin BGC and the assignment of Region 2.9 as the burriogladiodin BGC were supported by BLASTN comparison to reference loci.

Table S4. Overview of ^{1}H (600 MHz) and ^{13}C (150 MHz) NMR signals for gladiostatin in CD_3OD.

No.	δ_H (multiplicity, J = Hz)	δ_C (type)
1	-	175.5 (C)
1′	-	175.4 (C)
2	2.34 (overlapping), 2.72 (overlapping)	38.0 (CH_2)
2′	2.36 (overlapping), 2.65 (overlapping)	39.2 (CH_2)
3	2.34 (overlapping)	28.7 (CH)
4	1.43 (m), 1.51 (ddd, 13.8, 9.5, 3.9)	42.8 (CH_2)
5	4.13 (m)	65.7 (CH)
6	2.60 (dd, 16.9, 5.0), 2.71 (dd, 16.9, 7.6)	49.6 (CH_2)
7	-	212.6 (C)
8	3.22 (m)	51.7 (CH)
8-Me	1.11 (d, 6.9)	16.4 (CH_3)
9	5.38 (ddt, 15.3, 8.5, 1.4)	130.4 (CH)
10	5.64 (dtd, 15.3, 6.9, 1.0)	134.9 (CH)
11	2.05 (m, 2H)	33.6 (CH_2)
12	1.38 (m, 2H)	30.5 (CH_2)
13	1.31–1.34 (overlapping, 2H)	30.4 (CH_2)
14	1.31–1.34 (overlapping, 2H)	30.3 (CH_2)
15	1.31–1.34 (overlapping, 2H)	30.2 (CH_2)
16	1.31–1.34 (overlapping, 2H)	30.1 (CH_2)
17	1.60 (m, 2H)	24.4 (CH_2)
18	2.90 (br t, 7.3, 2H)	43.3 (CH_2)
19	-	199.1 (C)
20	-	127.4 (C)

21	-	173.6 (C)
1″	5.94 (s)	99.5 (CH)
2″	-	170.4 (C)
3″	2.30 (s)	13.6 (CH_3)

Table S5. Overview of ^{1}H (600 MHz) and ^{13}C (150 MHz) NMR signals for gladiostatin degradation product in pyridine-d_5.

No.	δ_H (multiplicity, J = Hz)	δ_C (type)
1		174.3 (C)
1′		174.2 (C)
2	2.49 (overlapping), 2.98 (dd, 16.5, 4.2)	38.3 (CH_2)
2′	2.53 (overlapping), 2.91 (16.5, 4.4)	39.5 (CH_2)
3	2.68 (m, 2H)	28.5 (CH)
4	1.59 (m), 1.75 (m)	43.1 (CH_2)
5	4.61 (m)	65.0 (CH)
6	2.75 (dd, 16.5, 4.4), 3.03 (dd, 16.5, 8.0)	49.8 (CH_2)
7		210.9 (C)
8	3.38 (m)	51.2 (CH)
8-Me	1.27 (d, 6.7)	16.7 (CH_3)
9	5.56 (dd, 15.3, 8.2)	130.1 (CH)
10	5.69 (dd, 15.3, 6.7)	133.9 (CH)
11	2.00 (m, 2H)	33.2 (CH_2)
12	1.31 (m, 2H)	30.0 (CH_2)
13	1.17–1.27 (overlapping, 2H)	29.9 (CH_2)
14	1.17–1.27 (overlapping, 2H)	29.8 (CH_2)
15	1.17–1.27 (overlapping, 2H)	29.7 (CH_2)
16	1.17–1.27 (overlapping, 2H)	29.7 (CH_2)

17	1.60 (m, 2H)	24.4 (CH_2)
18	2.49 (overlapping, 2H)	43.1 (CH_2)
19		206.7 (C)
20	3.49 (s, 2H)	42.4 (CH_2)
21	--	--
1″	9.65 (s)	194.2 (CH)
2″		144.9 (C)
3″	6.18 (s), 6.35 (s)	137.3 (CH_2)

Table S6. Overview of ^1H (600 MHz) and ^{13}C NMR signals (150 MHz) for gladiolinen in CD_3OD.

No.	Atom type	^1H(ppm)	^{13}C(ppm)
1	CO	-	173.7
2	CH_2	2.57, 2.43 (2x1H,2xm)	41.0
3	CH	4.09 (1H, m)	71.5
4	CH	1.62 (1H, m)	45.0
4-Me	CH_3	0.92 (3H, m)	10.4
5	CH	3.94 (1H, m)	71.7
6	CH_2	1.65 (2H, m)	39.8
7	CH	3.71 (1H, m)	73.4
8	CH	1.72 (1H, m)	38.7
8-Me	CH_3	0.91, (3H, d, 6.9)	14.3
9	CH_2	2.26, 2.05 (2x1H,2xm)	37.2
10	CH	5.73 (1H, dt, 7.0Hz, 15.3Hz)	133.7
11	CH	6.36 (1H, dd, 10.1Hz, 15 Hz)	128.2
12	CH	6.02 (1H, m)	131.5
13	CH	5.34 (1H, m)	126.2
14	CH_2	2.44, 2.35 (2x1H.2xm)	32.1
15	CH	3.26 (1H, m)	82.5

15-OMe	CH_3	3.34 (3H, s)	56.9
16	CH_2	1.46 (2H, m)	33.9
17	CH_2	1.46, 1.34 (2 x1H,2xm)	22.6
18	CH_2	1.45,1.35 (2x1H,2xm)	39.1
19	CH	3.53 (1H, m)	68.2
20	CH_2	1.66, 1.50 (2x1H, 2xm)	40.5
21	CH	5.13 (1H, dd, 5, 10.68)	75.7
22	C	-	129.9
22-Me	CH_3	1.02 (3H, d, 6.7)	16.7
23	CH	5.54 (1H, m)	129.9
24	CH	5.54 (1H, m)	139.2
25	C	-	139.2
25-Me	CH_3	1.24 (3H, d, 4.08)	27.9
26	CH_2	1.56 (2H, m)	43.4
27	CH_2	2.09 (2H, m)	28.5
28	CH	5.58 (1H, m)	133.7
29	CH	6.04 (1H, m)	131.4
30	CH	6.03 (1H, m)	134.0
31	CH	5.57 (1H, m)	129.1

32	CH_2	2.19 (2H, m)	42.6
33	CH	3.66 (1H, m)	70.0
34	CH_2	1.41, 1.20 (2x1H, 2xm)	45.0
35	CH	1.70 (1H, m)	30.1
35-Me	CH_3	0.91 (3H, m)	19.2
36	CH_2	1.62, 1.46 (2x1H, 2xm)	34.0
37	CH_2	2.30 (2H, m)	32.9
38	C	-	178.1
Solvent peak	Ethanol-CH2	3.61 (2H, q)	58.4
Solvent peak	Ethanol-CH3	1.17 (3H, t)	18.3
Solvent peak	MeOD	3.31	49.0

Table S7. Fold changes of the gene transcripts in BGC for gladiostatin upon disruption of *lttr_0390.*

Protein Stable ID	Gene description	Gene name	Amino acids	Locus[a]	log_2FC (Δ0390/WT)	log_2FC[b] ($\Delta 0390_{com}/\Delta 0390$)
WP_186079658.1	histidine phosphatase family protein	JKG63_RS34710	233	NZ_CP068050.1: 3868618-3869319(+)	3.463	-3.088
WP_236594001.1	ACP S-malonyltransferase	JKG63_RS34715 (fabD)	1111	NZ_CP068050.1: 3869419-3872754(+)	4.494	-2.727
WP_036055451.1	phosphopantetheine-binding protein	JKG63_RS34720	80	NZ_CP068050.1: 3872796-3873038(+)	4.036	-1.626
WP_036055452.1	asparagine synthase (glutamine-hydrolyzing)	JKG63_RS34725 (asnB)	665	NZ_CP068050.1: 3873133-3875130(+)	5.06	-3.758
WP_201446874.1	SDR family NAD(P)-dependent oxidoreductase	JKG63_RS34730	3247	NZ_CP068050.1: 3875123-3884866(+)	5.44	-3.738
WP_201446875.1	SDR family NAD(P)-dependent oxidoreductase	JKG63_RS18610	10170	NZ_CP068050.1: 3884900-3915412(+)	6.002	-4.288
WP_186099308.1	HAD family hydrolase	JKG63_RS18615	237	NZ_CP068050.1: 1706-2419(+)	5.275	-3.452
WP_042287319.1	NADP-dependent oxidoreductase	JKG63_RS18620	340	NZ_CP068050.1: 2454-3476(+)	4.842	-4.701

a: NCBI Reference Sequence NZ_CP068050.1 represents chromosome 2 of the BBB-01 strain.

b: $\Delta 0390_{com}$ denotes the complemented strain Δ0390/pBBR-0390.

Table S8. Fold changes of the gene transcripts in BGC for haereogladiodins upon disruption of *lttr_0390.*

Protein Stable ID	Gene description	Gene name	Amino acids	Locus[a]	log_2FC (Δ0390/WT)	log_2FC[b] ($Δ0390_{com}$/Δ0390)
WP_042285834.1	VOC family protein	JKG63_RS23420	183	NZ_CP068050.1: 1123906-1124457(+)	4.315	-1.767
WP_201447059.1	aminodeoxychorismate synthase component I	JKG63_RS23425 pabB	522	NZ_CP068050.1: 1124608-1126176(+)	4.671	-1.778
WP_201447061.1	non-ribosomal peptide synthetase	JKG63_RS23430	5647	NZ_CP068050.1: 1126276-1143219(+)	4.092	-2.091
WP_201447063.1	cupin-like domain-containing protein	JKG63_RS23435	394	NZ_CP068050.1: 1143236-1144420(+)	4.078	-2.285
WP_201447065.1	MFS transporter	JKG63_RS23440	424	NZ_CP068050.1: 1144417-1145691(+)	3.99	-1.266

a: NCBI Reference Sequence NZ_CP068050.1 represents chromosome 2 of the BBB-01 strain.

b: $Δ0390_{com}$ denotes the complemented strain Δ0390/pBBR-0390.

Table S9. Fold changes of the gene transcripts in an unpredicted BGC, presumably for N-acyl amino acid derivatives, upon disruption of *lttr_0390.*

Protein Stable ID	Gene description	Gene name	Amino acids	Locus[a]	log_2FC (Δ0390/WT)	log_2FC[b] ($Δ0390_{com}$/Δ0390)
WP_186211811.1	alkaline phosphatase family protein	JKG63_RS18895	786	NZ_CP068050.1: 64658-67018(-)	4.057	0.764
WP_226284752.1	iron-containing redox enzyme family protein	JKG63_RS18905	221	NZ_CP068050.1: 68356-69021(+)	4.144	-0.732
WP_046580544.1	N-acyl amino acid synthase FeeM domain-containing protein	JKG63_RS18910	243	NZ_CP068050.1: 69328-70059(+)	4.256	-1.624
WP_025097820.1	hypothetical protein	JKG63_RS18915	237	NZ_CP068050.1: 70063-70776(+)	4.16	-1.892
WP_230676465.1	DUF2817 domain-containing protein	JKG63_RS18920	369	NZ_CP068050.1: 70776-71885(+)	4.299	-1.715
WP_201446879.1	threonine/serine dehydratase	JKG63_RS18925	343	NZ_CP068050.1: 71905-72936(+)	4.173	-1.707
WP_013691251.1	DMT family transporter	JKG63_RS18930	298	NZ_CP068050.1: 72986-73882(+)	3.75	-2.660

a: NCBI Reference Sequence NZ_CP068050.1 represents chromosome 2 of the BBB-01 strain.

b: $Δ0390_{com}$ denotes the complemented strain Δ0390/pBBR-0390.

Table S10. Fold changes of the gene transcripts in a cluster encoding cytochrome bd quinol oxidase upon disruption of *lttr_0390.*

Protein Stable ID	Gene description	Gene name	Amino acids	Locus	log_2FC (Δ0390/WT)	log_2FC ($Δ0390_{com}$/Δ0390)
WP_013699432.1	cytochrome oxidase putative small subunit (cydP)	JKG63_RS16695	89	NZ_CP068049.1: 3777107-3777376(+)	2.845	-3.330
WP_036049121.1	cytochrome ubiquinol oxidase subunit I	JKG63_RS16700	523	NZ_CP068049.1: 3777366-3778937(+)	3.615	-3.893
WP_036049120.1	cytochrome d ubiquinol oxidase subunit II (cydB)	JKG63_RS16705	378	NZ_CP068049.1: 3778975-3780111(+)	4.225	-5.119
WP_013699435.1	cytochrome bd-I oxidase subunit (cydX)	JKG63_RS16710	39	NZ_CP068049.1: 3780178-3780297(+)	4.542	-22.490

a: NCBI Reference Sequence NZ_CP068049.1 represents chromosome 1 of the BBB-01 strain.

b: $Δ0390_{com}$ denotes the complemented strain Δ0390/pBBR-0390.

Table S11. Fold changes of the gene transcripts in BGC for sinapigladioside upon disruption of *lttr_0390.*

Protein Stable ID	Gene description	Gene name	Amino acids	Locus[a]	log_2FC (Δ0390/WT)	log_2FC[b] ($Δ0390_{com}$/Δ0390)
WP_103691304.1	GlxA family transcriptional regulator	JKG63_RS09085	314	NZ_CP068049.1: 2001738-2002682(+)	-1.053	3.596
WP_052747423.1	isocyanide synthase family protein	JKG63_RS09090	327	NZ_CP068049.1: 2003450-2004433(+)	-5.448	7.423
WP_172878250.1	Bcr/CflA family efflux MFS transporter	JKG63_RS09095	402	NZ_CP068049.1: 2004462-2005670(+)	-5.097	4.485
WP_046579374.1	protein tyrosine phosphatase family protein	JKG63_RS09100	156	NZ_CP068049.1: 2005700-2006170(+)	-5.523	8.923
WP_046579373.1	L-tyrosine/L-tryptophan isonitrile synthase family protein	JKG63_RS09105	611	NZ_CP068049.1: 2006167-2008002(+)	-5.283	6.585
WP_186009781.1	glycosyltransferase	JKG63_RS09110	429	NZ_CP068049.1: 2008028-2009317(+)	-4.84	4.516
WP_201446556.1	hypothetical protein	JKG63_RS35570	811	NZ_CP068049.1: 2009424-2011859(+)	-4.616	4.987
WP_046579369.1	class I SAM-dependent methyltransferase	JKG63_RS09120	226	NZ_CP068049.1: 2011870-2012550(+)	-4.671	5.250
WP_186160216.1	UbiA family prenyltransferase	JKG63_RS09125	472	NZ_CP068049.1: 2012568-2013986(+)	-4.747	5.172
WP_186103755.1	FAD-binding oxidoreductase	JKG63_RS09130	460	NZ_CP068049.1: 2013983-2015365(+)	-4.027	5.548
WP_181278793.1	SDR family oxidoreductase	JKG63_RS09135	246	NZ_CP068049.1: 2015362-2016102(+)	-4.127	16.113
WP_046579363.1	NAD-dependent epimerase/dehydratase family protein	JKG63_RS09140	311	NZ_CP068049.1: 2016099-2017034(+)	-5.393	7.304
WP_186271675.1	NAD(P)/FAD-dependent oxidoreductase	JKG63_RS09145	431	NZ_CP068049.1: 2017045-2018340(+)	-4.756	4.370
WP_186172247.1	GtrA family protein	JKG63_RS09150	152	NZ_CP068049.1: 2018337-2018795(+)	-3.994	20.974

a: NCBI Reference Sequence NZ_CP068049.1 represents chromosome 1 of the BBB-01 strain.

b: $Δ0390_{com}$ denotes the complemented strain Δ0390/pBBR-0390.

Table S12. Fold changes of the gene transcripts in BGC for gladiolin upon disruption of *lttr_0390.*

Protein Stable ID	Gene description	Gene name	Amino acids	Locus[a]	$\log_2$FC (Δ0390/WT)	$\log_2$FC[b] (Δ0390$_{com}$/Δ0390)
WP_201446638.1	SDR family NAD(P)-dependent oxidoreductase, transAT-PKS	JKG63_RS27605	5901	NZ_CP068050.1: 2155008-2172713(-)	-5.986	2.588
WP_186100303.1	SDR family NAD(P)-dependent oxidoreductase	JKG63_RS27610	3620	NZ_CP068050.1: 2172805-2183667(-)	-5.805	2.869
WP_201446639.1	SDR family NAD(P)-dependent oxidoreductase	JKG63_RS27615	4983	NZ_CP068050.1: 2183693-2198644(-)	-6.237	3.004
WP_201446640.1	SDR family NAD(P)-dependent oxidoreductase	JKG63_RS27620	3623	NZ_CP068050.1: 2198658-2209529(-)	-5.939	2.855
WP_201446641.1	SDR family NAD(P)-dependent oxidoreductase	JKG63_RS27625	3803	NZ_CP068050.1: 2209517-2220928(-)	-5.874	3.402
WP_201446642.1	SDR family NAD(P)-dependent oxidoreductase	JKG63_RS27630	5461	NZ_CP068050.1: 2220925-2237310(-)	-5.479	3.082
WP_308732667.1	asparagine synthase (glutamine-hydrolyzing)	JKG63_RS27635 (asnB)	648	NZ_CP068050.1: 2237307-2239277(-)	-4.178	1.899
WP_052409166.1	ACP S-malonyltransferase	JKG63_RS27640	276	NZ_CP068050.1: 2239374-2240270(-)	-4.276	2.666
WP_036053930.1	acyl carrier protein	JKG63_RS27645	88	NZ_CP068050.1: 2240267-2240533(-)	-4.116	3.190
WP_157693238.1	hypothetical protein	JKG63_RS27650	76	NZ_CP068050.1: 2240528-2240758(+)	-3.715	1.641
WP_052409165.1	pfaD family polyunsaturated fatty acid/polyketide biosynthesis protein	JKG63_RS27655	463	NZ_CP068050.1: 2241370-2242761(+)	-5.380	4.302
WP_036053932.1	acyl carrier protein	JKG63_RS27660	82	NZ_CP068050.1: 2242764-2243012(+)	-5.057	4.824
WP_160294757.1	β-ketoacyl synthase N-terminal-like domain-containing protein	JKG63_RS27665	409	NZ_CP068050.1: 2242990-2244219(+)	-4.911	4.368
WP_036053936.1	hydroxymethylglutaryl-CoA synthase	JKG63_RS27670	420	NZ_CP068050.1: 2244239-2245501(+)	-5.027	2.909

WP_036053939.1	enoyl-CoA hydratase/isomerase	JKG63_RS27675	263	NZ_CP068050.1: 2245498-2246289(+)	-5.066	2.112
WP_036053941.1	polyketide synthase	JKG63_RS27680	249	NZ_CP068050.1: 2246289-2247038(+)	-4.689	2.923
WP_201446644.1	hypothetical protein	JKG63_RS27685	1078	NZ_CP068050.1: 2247055-2250291(+)	-4.725	2.811
WP_042285164.1	DUF4180 domain-containing protein	JKG63_RS27690	120	NZ_CP068050.1: 2250291-2250653(+)	-5.602	3.889
WP_052409164.1	amidase	JKG63_RS27695	467	NZ_CP068050.1: 2250666-2252069(+)	-4.888	2.178
WP_201446645.1	ACP S-malonyltransferase	JKG63_RS27700 (fabD)	395	NZ_CP068050.1: 2252176-2253363(+)	-4.835	3.001
WP_186049781.1	4'-phosphopantetheinyl transferase superfamily protein	JKG63_RS27705	285	NZ_CP068050.1: 2253330-2254187(-)	-3.668	2.120
WP_105826972.1	acyltransferase domain-containing protein	JKG63_RS27710	325	NZ_CP068050.1: 2254218-2255195(-)	-3.681	1.419
WP_330932717.1	MATE family efflux transporter	JKG63_RS27715	428	NZ_CP068050.1: 2255267-2256652(-)	-3.878	1.821

a: NCBI Reference Sequence NZ_CP068050.1 represents chromosome 2 of the BBB-01 strain.

b: $\Delta0390_{com}$ denotes the complemented strain Δ0390/pBBR-0390.

Table S13. Fold changes of the gene transcripts in icosalide BGC, centering on the non-ribosomal peptide synthetase encoding gene, upon *lttr_0390* disruption.

Protein Stable ID	Gene description	Gene name	Amino acids	Locus[a]	log_2FC (Δ0390/WT)	log_2FC[b] (Δ0390$_{com}$/Δ0390)
WP_036054925.1	MFS transporter	JKG63_RS31475	435	NZ_CP068050.1: 3126261-3127568(+)	0.793	1.641
WP_186081666.1	S8 family peptidase	JKG63_RS31485	512	NZ_CP068050.1: 3128168-3129706(+)	1.002	1.378
WP_186094351.1	Xaa-Pro dipeptidyl-peptidase	JKG63_RS31490	649	NZ_CP068050.1: 3129825-3131774(+)	0.597	-0.166
WP_236593904.1	DUF3304 domain-containing protein	JKG63_RS31500	229	NZ_CP068050.1: 3132250-3132939(+)	-0.243	1.361
WP_201446768.1	non-ribosomal peptide synthetase	JKG63_RS31505	5011	NZ_CP068050.1: 3133128-3148163(-)	-4.334	5.605
WP_186049345.1	diguanylate cyclase	JKG63_RS31515	383	NZ_CP068050.1: 3148801-3149952(+)	-0.387	1.462
WP_201446769.1	helix-turn-helix domain-containing protein	JKG63_RS31520	239	NZ_CP068050.1: 3149989-3150708(-)	0.268	17.084

a: NCBI Reference Sequence NZ_CP068050.1 represents chromosome 2 of the BBB-01 strain.

b: Δ0390$_{com}$ denotes the complemented strain Δ0390/pBBR-0390.

Table S14. Fold changes of the gene transcripts related to motility and biofilm formation.

Protein Stable ID	Gene description	Gene name	log_2FC (Δ0390/WT)	p-value[a]
**Motility**				
WP_013690705.1	flagellar motor stator protein MotA	JKG63_RS21660	-2.482	0.00035
WP_186047795.1	flagellar motor protein MotB	JKG63_RS21655	-2.255	0.0012
**Biofilm formation**				
WP_036055860.1	fimbrial protein	JKG63_RS22080	-2.226	0.0012
WP_160293328.1	pilus assembly protein PilN	JKG63_RS01800	-2.226	0.0012
WP_013696399.1	pilus assembly protein PilM	JKG63_RS01795	-1.502	0.0758
WP_160294077.1	fimbria/pilus outer membrane usher protein	JKG63_RS22090	-1.804	0.0135
WP_013690663.1	fimbria/pilus periplasmic chaperone	JKG63_RS22085	-1.404	0.0486

a: only the fold change in the expression of MotA has a q value less than 0.05; the rest have q values more than 0.05.

References

(1) Lin, Y. T.; Lee, C. C.; Leu, W. M.; Wu, J. J.; Huang, Y. C.; Meng, M. Fungicidal activity of volatile organic compounds emitted by *Burkholderia gladioli* strain BBB-01. *Molecules* **2021**, *26* (3), 745. DOI: 10.3390/molecules26030745.

(2) Veselova, M. A.; Romanova, Y. M.; Lipasova, V. A.; Koksharova, O. A.; Zaitseva, Y. V.; Chernukha, M. U.; Gintsburg, A. L.; Khmel, I. A. The effect of mutation in the clpX gene on the synthesis of N-acyl-homoserine lactones and other properties of *Burkholderia cenocepacia* 370. *Microbiol Res* **2016**, *186-187*, 90-98. DOI: 10.1016/j.micres.2016.03.009.
